# A Crucial Role for Infected-Cell/Antibody Immune Complexes in the Enhancement of Endogenous Antiviral Immunity by Short Passive Immunotherapy

**DOI:** 10.1371/journal.ppat.1000948

**Published:** 2010-06-10

**Authors:** Henri-Alexandre Michaud, Tiphanie Gomard, Laurent Gros, Kevin Thiolon, Roudaina Nasser, Chantal Jacquet, Javier Hernandez, Marc Piechaczyk, Mireia Pelegrin

**Affiliations:** 1 Institut de Génétique Moléculaire de Montpellier, UMR 5535 CNRS, Montpellier, France; 2 Université Montpellier 2, Montpellier, France; 3 Université Montpellier 1, Montpellier, France; National Institutes of Health-NIAID, United States of America

## Abstract

Antiviral monoclonal antibodies (mAbs) represent promising therapeutics. However, most mAbs-based immunotherapies conducted so far have only considered the blunting of viral propagation and not other possible therapeutic effects independent of virus neutralization, namely the modulation of the endogenous immune response. As induction of long-term antiviral immunity still remains a paramount challenge for treating chronic infections, we have asked here whether neutralizing mAbs can, in addition to blunting viral propagation, exert immunomodulatory effects with protective outcomes. Supporting this idea, we report here that mice infected with the FrCas^E^ murine retrovirus on day 8 after birth die of leukemia within 4–5 months and mount a non-protective immune response, whereas those rapidly subjected to short immunotherapy with a neutralizing mAb survive healthy and mount a long-lasting protective antiviral immunity with strong humoral and cellular immune responses. Interestingly, the administered mAb mediates lysis of infected cells through an antibody-dependent cell cytotoxicity (ADCC) mechanism. In addition, it forms immune complexes (ICs) with infected cells that enhance antiviral CTL responses through FcγR-mediated binding to dendritic cells (DCs). Importantly, the endogenous antiviral antibodies generated in mAb-treated mice also display the same properties, allowing containment of viral propagation and enhancement of memory cellular responses after disappearance of the administered mAb. Thus, our data demonstrate that neutralizing antiviral mAbs can act as immunomodulatory agents capable of stimulating a protective immunity lasting long after the end of the treatment. They also show an important role of infected-cells/antibody complexes in the induction and the maintenance of protective immunity through enhancement of both primary and memory antiviral T-cell responses. They also indicate that targeting infected cells, and not just viruses, by antibodies can be crucial for elicitation of efficient, long-lasting antiviral T-cell responses. This must be considered when designing antiviral mAb-based immunotherapies.

## Author Summary

Monoclonal antibodies (mAbs) constitute the largest class of bio-therapeutic proteins and are increasingly being considered as drugs to fight both acute and chronic severe human viral diseases. Most antiviral mAb-based treatments conducted so far, whether in humans or in animal models, have only considered the blunting of viral propagation through direct virus neutralization. However, mAbs might also operate via complementary mechanisms owing to their ability to interact with various components of the immune system. Using a lethal mouse model of retrovirally-induced leukemia, we report here that a neutralizing mAb administered to infected mice for a short period of time can, in addition to its direct effect on viral spread, induce a strong, long-lasting antiviral immune response protecting mice from disease development long after the end of the treatment. Although the initiation and maintenance of this long-term immunity is multi-factorial, we demonstrate a crucial role for the immune complexes formed between antiviral antibodies and infected cells in this process. Our work reveals a thus far underappreciated vaccine-like effect of antiviral neutralizing mAbs, which will have to be considered for future treatment of life-threatening viral infections.

## Introduction

Monoclonal antibodies (mAbs) constitute the largest class of biomedical proteins [1]. They are increasingly being considered as therapeutic agents to fight both acute and chronic severe human viral diseases, with many of them developed in the most recent period [1], [2]. Non exclusively, one can cite neutralizing mAbs against Ebola- [3], West Nile (WNV)- [4], cytomegalo (CMV)- [5], avian H5N1- [6] and human influenza- [6], [7], severe acute respiratory syndrome (SARS)- [8], respiratory syncytial (RSV)- [9], hepatitis B (HBV)- [10], hepatitis C (HCV)- [11] and human immunodeficiency virus (HIV) [1], [12] that have shown antiviral activity in preclinical studies. One of these (Pavilizumab), directed against RSV, is commercially available, whereas others (see [2] for more information and references) are currently being tested in clinical studies for treating either acute cytopathic infections by WNV and CMV or severe chronic infections by HBV, HCV and HIV, which figure amongst the heaviest health burdens worldwide.

Most antiviral mAb-based treatments conducted so far, whether in humans or in animal models, have only considered the blunting of viral propagation through direct virus neutralization. However, mAbs might also operate via complementary mechanisms owing to their ability to interact with various components of the immune system via the effector functions borne by their constant regions. For example, certain viral antigens expressed at the surface of infected cells, such as envelope glycoproteins (Env) of immunodeficiency-inducing viruses, like HIV, or of cancer-inducing viruses, like HCV or murine oncoretroviruses, may also permit mAbs to target virus reservoirs via complement-dependent- (CDC) or antibody-dependent cell cytotoxicity (ADCC) mediated by natural killer cells. Moreover, mAbs form immune complexes (ICs) with viral antigens exposed at the surface of virions as well as on that of infected cells for certain viruses. As uptake, processing and MHC presentation of antigens by antigen-presenting cells (APCs) are significantly different depending on whether they are complexed or not to antibody, this may ultimately impact on endogenous immunity [13], [14]. There are several reasons why such alternative modes of action of mAbs have been poorly investigated *in vivo* to date. First, for technical reasons, the antiviral activity of certain mAbs has only been tested in immunodeficient preclinical animal models. Second, certain antiviral mAbs originating from one species have been tested in preclinical models from other species where constant region-dependent antibody functions are not, or poorly, preserved. Third, in addition to the paucity of antiviral mAbs in clinical trials, technical and ethical limitations prevent certain investigations in human beings. Establishing whether, and how, mAb-based treatments can orientate antiviral immune responses towards long-term protective immunity lasting long after the mAb has disappeared is, however, important as this might influence the design of mAb immunotherapies of severe chronic life-threatening viral infections. To address this issue, we have resorted to the infection of immunocompetent mice by the FrCas^E^ retrovirus, as it is one of the rare model systems permitting extensive analysis of the endogenous immune response after passive mAb-based immunotherapy under conditions of both chronic infection and pathological development [15]–[17].

FrCas^E^ is an ecotropic retrovirus derived from the Friend murine leukemia virus (MuLV) [15]. Upon inoculation to newborn mice under the age of 5 days, it first propagates in lymphoid organs before penetrating into the central nervous system (CNS), which leads to a fatal neurodegeneration within 1–2 months [17]–[19]. In contrast, animals infected at a later stage do not develop any neurological illness due to the inability of FrCas^E^ to penetrate the CNS after day 8 after birth but, instead, may develop fatal erythroleukemia (see below). We have recently shown that, when infected newborn mice (day 3 after birth) are briefly treated by the neutralizing IgG2a/κ mAb 667 [17], [20], [21] shortly after infection, all animals survive and show no signs of neurodegeneration or leukemia for more than 1 year [18], [22], [23]. Survival in good health is not due exclusively to an immediate effect on the viral load preventing brain infection. It also depends on the development of an endogenous long-term protective antiviral immunity that both contains residual viral replication after clearance of 667 and permits mice to resist to viral challenges. This immunity is a typical Th1 response comprising a strong cytotoxic T-cell response against FrCas^E^-infected cells [22] and a potent anti-FrCas^E^ humoral response principally made up of IgG2a [18], [24]. Moreover, it is rapidly mounted after mAb treatment and contrasts with the non-protective responses usually developing in MuLV-infected mice eventually dying from virally-induced diseases [25,26 and this work]. This observation is important as it suggests that, in case of suspicion of infection by life-threatening viruses such as HIV or HCV, rapid administration of mAb-based immunotherapies before the natural antiviral response is initiated, may favor the mounting of a potent protective immunity. This is all the more to be considered that strong neutralizing antibody responses in the early phase of infection usually correlate with better long-term control of various persisting viruses [27]–[29]. However, only a small fraction of infected individuals develop such responses as most persistent viruses induce delayed and weak virus neutralizing antibody responses that correlate with the establishment of chronic infection [5], [30]. Taking this into account, it is now crucial to understand how the endogenous protective antiviral response is generated in mAb-treated animals, why it is so long lasting and whether administered mAbs actually exert immunomodulatory actions. Addressing these issues in the FrCas^E^ system requires detailed comparison of the antiviral immune responses developing in 667-treated and -non-treated infected mice, which was not possible in our original acute neonatal infection model because simply infected mice cannot mount endogenous immune responses due to rapid health deterioration (unpublished data). We therefore developed here a chronic infection setting using older mice, that leads to death of 100% of the animals by erythroleukemia within 4–5 months, which provides sufficient time for informative comparison of the two types of immune responses. Our data clearly point to immunomodulatory effects of the administered mAb. Moreover, they reveal an important mechanism whereby antiviral immunity is initiated and maintained in the long-term via the formation of immune complexes with infected cells. Our study has consequences for the design of future antiviral immunotherapies.

## Results

### Transient 667 mAb treatment efficiently limits viral propagation and prevents mice from developing FrCas^E^-induced erythroleukemia

**Figure 1:**
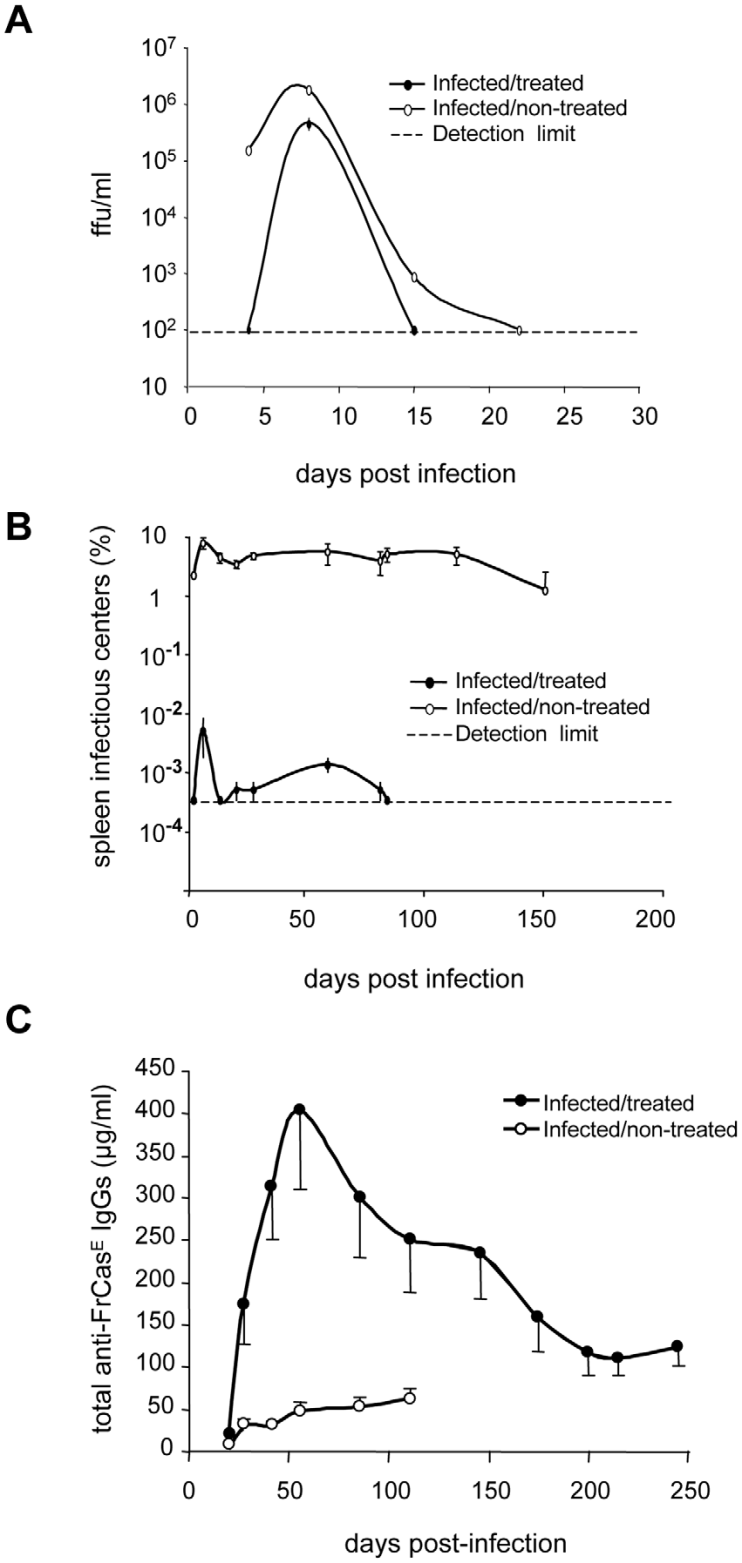
Differences in viremia, spleen infectious centers and anti-FrCas^E^ IgG responses between infected/treated and infected/non-treated mice. *Serum viremia*, presented as focus-forming units (ffu)/ml (A) and *spleen infectious centers* (B) were assayed as described in the Methods section. Two groups of 8 day-old mice were infected with FrCas^E^. One group was treated with 667 (infected/treated) 1 hour post-infection and on days 2 and 5 post-infection and the other was not treated (infected/non-treated). A group of mice that was not infected but that was treated with 667 (non-infected/treated), was taken as a negative control (not shown). The data presented are representative of 2 independent experiments. Each time point is the average of values obtained from 2 animals per time point except in the case of day 8 (5 animals). Non-infected/treated mice were negative for both serum viremia and SICs and are not presented. Error bars indicate SEM. *Total anti-FrCas^E^ IgG*s (C) were assayed by ELISA. Mice were infected and treated as in A and B. One of 2 independent experiments with similar outcomes is presented. The data are the average of values obtained from at least 10 animals per time point. Non-infected/treated mice were negative for anti-FrCas^E^ IgGs and are therefore not presented. Error bars indicate SEM.

First, we created a model of chronic infection by FrCas^E^ leading to the death of 100% of mice by erythroleukemia with clear and synchronous symptomatology. This was achieved by virus inoculation on day 8 after birth. All mice showed the first symptoms of erythroleukemia at 2 months post-infection. These were spleen swelling, due to exacerbated erythroblast proliferation, and reduction of hematocrits, resulting from combined blockade of erythropoiesis of infected erythroblasts and exhaustion of non-infected red blood cells [26]. Mice usually died between day 120–150 with very low hematocrits (36%±1 versus 50%±1 in control animals) and dramatically enlarged spleens (up to 18-fold increase in weight). We then showed that a short 667 mAb treatment prevents infected animals from developing erythroleukemia by comparing FrCas^E^-infected, 667 mAb-treated (infected/treated) mice to 3 control groups: (i) infected mice not treated with 667 (infected/non-treated), (ii) infected mice treated with an isotype-matched control mAb (anti-β-2,6-fructosan IgG2a mAb) (infected/control mAb-treated mice) and (iii) non-infected mice receiving 667 (non-infected/treated). 667 was administered 3 times: 1 hour after virus inoculation, which is a time sufficient for the establishment of infection (see ref. [31] and below), and then 2- and 5 days later (infected/treated mice). Importantly, under these conditions, 667 became undetectable within less than 20–25 days post-injection. All non-infected/treated mice and most infected/treated mice (19 out of 21) survived without pathological symptoms for the 8 months of the experiments whereas all infected/non-treated- and all infected/control mAb-treated mice died of erythroleukemia by 4–5 months post-infection (**Figure S1**). Finally, as spleen is a major organ for MuLV replication, we assessed to which extent 667 could affect viral propagation by assaying both serum viremia (**Figure 1A**) and the number of infected splenocytes (spleen infectious centers or SICs) (**Figure 1B**). In infected/non-treated animals, viremia was transient, peaking (1.7×10^6^ focus-forming units- or ffu/ml) by day 8 post-infection with levels that were already high on day 4 post-infection and return to undetectable levels by day 20 (limit of detection: 10^2^ ffu/ml). In infected/treated animals, this peak was reduced by approximately 4-fold and was narrower (viremia was undetectable on day 4 post-infection and returned to an undetectable level earlier than day 20). The effect of 667 was more marked on SICs than on viremia as infected/non-treated mice showed a high and constant level of SICs (2–5%) up to death or experiment termination (day 150) whereas SICs were reduced by 1000-fold from the earliest time points tested onwards and became undetectable after day 80 (limit of detection: 1 SIC per 3×10^5^ splenocytes) in infected/treated animals. Importantly, no significant rebound of viral propagation, as assayed by SICs, was observed in infected/treated mice after 20–25 days post-infection. This suggested the existence of indirect mechanisms induced by the mAb treatment for reduction/elimination of retrovirus-producing cells after 667 was cleared off from infected/treated mice.

### Infected/treated mice mount stronger humoral and CTL anti-FrCas^E^ immune responses than infected/non-treated mice

Next, we showed that infected/treated mice developed a stronger IgG response than infected/non-treated ones (**Figure 1C**). In these experiments, infected/non-treated animals displayed stable, but relatively weak (30–60 µg/ml), anti-FrCas^E^ total IgG levels until death or sacrifice of severely leukemic animals. In contrast, antiviral IgGs accumulated to much higher levels in infected/treated mice with a peak (400 µg/ml) by day 56 post-infection and a still high level (120 µg/ml) at the end of the experiment (day 243 post-infection). Although anti-FrCas^E^ IgG1 and -IgG2a were found in both groups of animals, IgG2a were always at least 2-fold more abundant and their predominance increased with time in infected/treated animals (**Figure S2A**), suggesting a prominent Th1 T-cell response (also see below). Finally, we observed that FrCas^E^ -neutralizing antibody titers were up to 4 to 8-fold higher in infected/treated mice than in infected/non-treated ones, indicating that neutralization activity in the two groups of animals is roughly proportional to the amount of anti-FrCas^E^ antibodies (**Figure S2B**).

**Figure 2:**
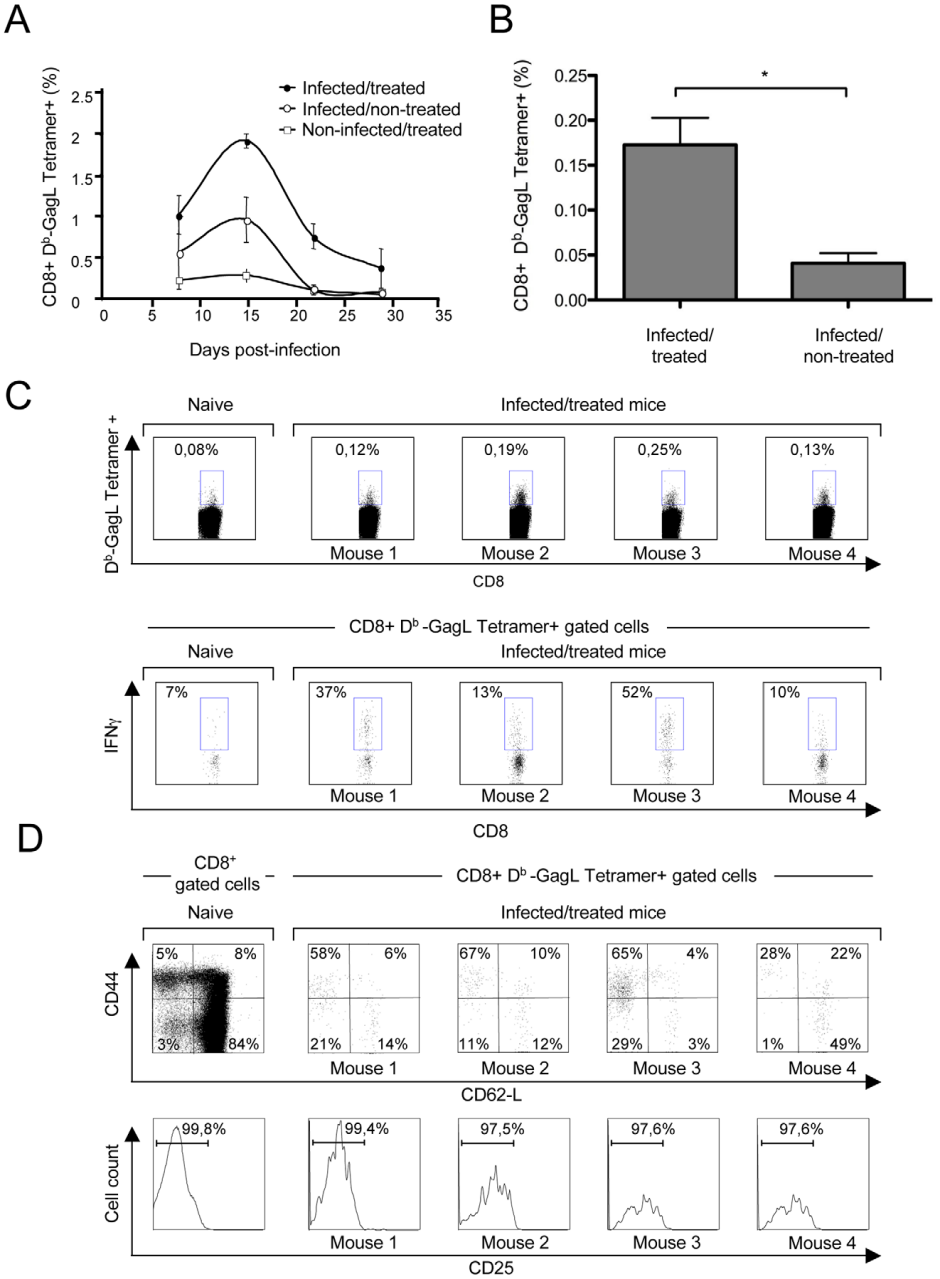
Primary CD8^+^ T-cell responses against FrCas^E^-infected cells. Mice were infected and treated, or not, as in **Figure 1**. (A) *Kinetics of the primary anti-FrCas^E^ CTL response*. Spleen cells from 2 mice of each group per time point (days 8, 22 and 28 post-infection) and from 5 mice per group at day 15 post-infection were stained with an anti-CD8 mAb and the D^b^-GagL tetramer and analyzed by flow cytometry. (B–D) *Phenotypic characterization of primary CD8^+^ T-cell responses in infected/treated animals.* Mice were infected and treated as in **Figure 1**. CD3*^+^* cells were isolated by negative selection from the spleens of 4 infected/treated- and 2 infected/non-treated mice on day 56 post-infection. Half of the CD3*^+^* cells were then stained with the D^b^-GagL tetramer and anti-CD8, anti-CD44, anti-CD62L and anti-CD25 mAb and analyzed by flow cytometry. In parallel, the other half of the CD3*^+^* cells were incubated with PMA-ionomycin plus brefeldin A and IFN-γ production by tetramer^+^CD8*^+^* T cells was assessed by intracellular staining. The statistical significance of data between infected/treated- and infected/non-treated mice was established using the Student's t test (*P = 0,045). (B) *Assay of GagL-specific CD8^+^ T cells in infected/treated and infected/non-treated mice on day 56 post-infection.* (C–D) *Phenotypic characterization of GagL-specific CD8^+^ T cells on day 56 post-infection.* Data from 1 age-matched non-infected/non-treated (naive) mouse and 4 infected/treated mice are presented.

We then compared the cellular immune responses against FrCas^E^-infected cells developing in infected/non-treated and infected/treated animals. To this end, we first assessed the primary CD8^+^ T-cell response against FrCas^E^-infected cells during the first month post-infection by flow cytometry analysis of splenic CD8^+^ T lymphocytes using a MHC class I (MHC I) H2-D^b^ tetramer loaded with the GagL peptide (D^b^-GagL tetramer) [32]. GagL is an immunodominant H2D^b^-restricted epitope conserved among the different MuLVs strains, which is derived from the leader region of a glycosylated transmembrane form of the Gag precursor polyprotein (gPr80^gag^). Importantly, gPr80^gag^ is expressed by infected cells but not incorporated into virions [33]. Both infected/non-treated and infected/treated mice developed a transient primary CD8^+^ T-cell response already detectable by day 8 post-infection and peaking by day 15 (**Figure 2A**). However, it was 2-fold higher in the latter group of mice at the peak of the response, which was also consistent with the time of SICs reduction in these mice (**Figure 1B**). In infected/non-treated animals, the CD8^+^ T-cell response dropped to background levels by day 22 post-infection (**Figure 2A**) whereas it was still detectable in infected/treated mice for at least 56 days, as evidenced by quantification of D^b^-GagL tetramer^+^ cells (**Figures 2B and 2C**). We also analyzed the phenotype of the GagL peptide-specific CD8^+^ T-cell population remaining after the contraction phase. At day 56 post-infection, most of the D^b^-GagL tetramer^+^ CD8^+^ T cells from infected/treated mice were CD44^hi^ (mean 65%), whereas only 13% of the CD8^+^ T cells in non-infected mice displayed this phenotype (**Figure 2D**). Moreover, the majority of the CD44^hi^ cells were CD62L^lo^ (**Figure 2D**). Interestingly, the assessment of CD25- and CD127 expression revealed that D^b^-GagL tetramer^+^ CD8^+^ T cells were CD25^lo^ and CD127^hi^ (**Figure 2D** and **Figure S3**). Finally, D^b^-GagL tetramer^+^ cells demonstrated effector functions, as revealed by the production of IFN-γ (**Figure 2C**). Taken together, these results show that infected/treated mice generated an important CD8^+^ T cell memory pool mainly comprising effector-memory cells.

**Figure 3:**
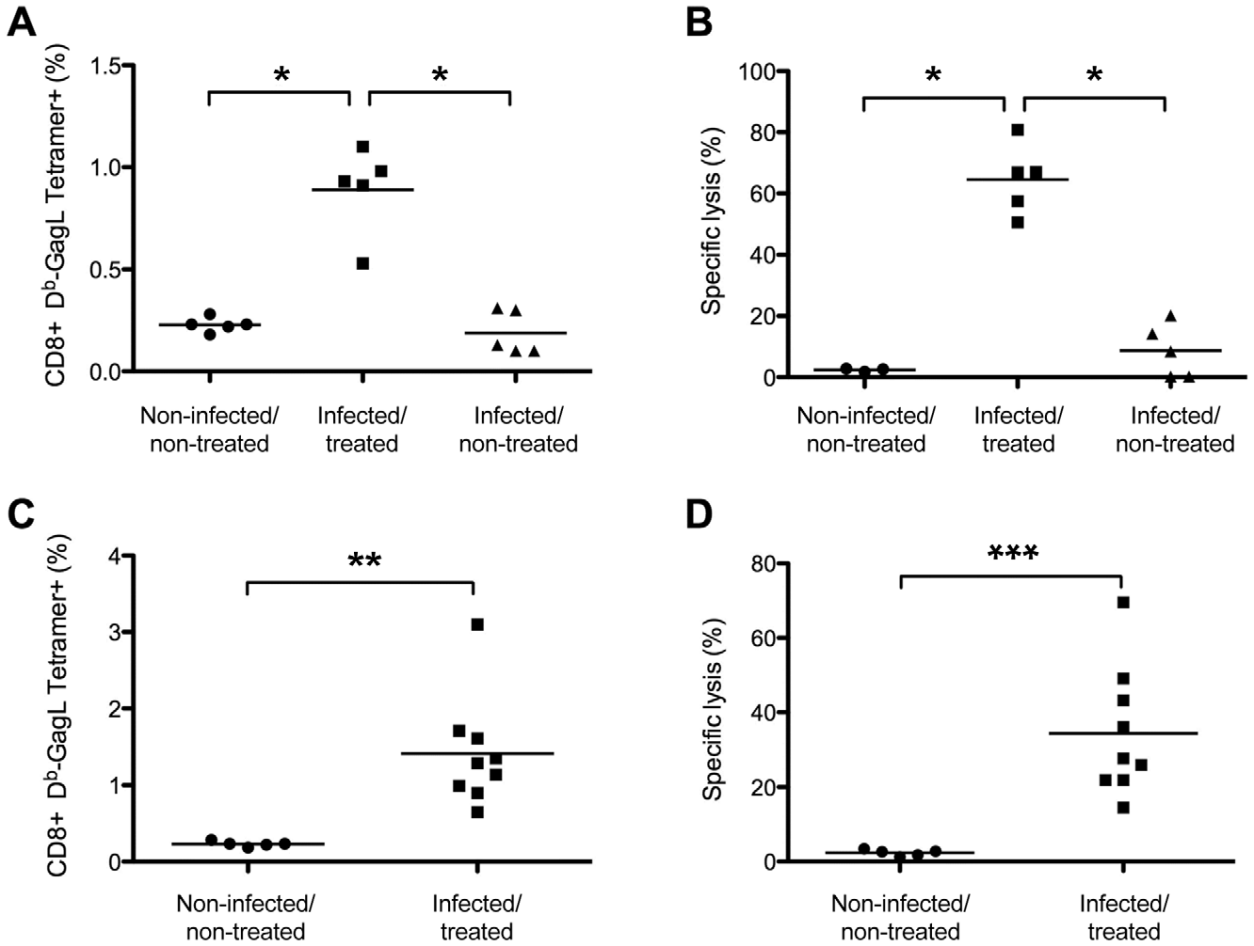
Long-term CTL memory responses in infected/treated animals versus control mice. Mice were challenged with FrCas^E^-infected splenocytes on days 56 or 116 post-infection and 9 days later CD8^+^ T-cell responses were assessed by measuring both the percentage of GagL-specific CD8^+^ T cells and specific *in vivo* CTL activity as described in the Methods section. (A–B) *Assay of GagL-specific memory CTL responses in infected/treated and infected/non-treated mice challenged on days 56 or 116 post-infection*. Infected/treated and infected/non-treated mice were challenged on day 56 (3 mice per group) or on day 116 (2 mice per group). Non-challenged age-matched non-infected/non-treated mice (3 on day 56 and 2 on day 116) were used as controls. The statistical significance of data between the infected/treated group and the other two groups was established using a non-parametric one-way ANOVA test with Dunn's multiple comparison post-test (*P<0,05). (C–D) *Assay of GagL-specific memory CTL responses in infected/treated mice challenged at month 8 post-infection*. Eight month-old infected/treated mice were challenged with FrCas^E^-infected splenocytes. Non-challenged aged-matched non-infected/non-treated mice were used as controls. Statistical significance of data between infected/treated- and non-infected/non-treated mice was established using the Student's t test (**P = 0,0034; *** *P* = 0,0015). Bars indicate mean values.

We then assessed the ability of GagL-specific memory CD8^+^ T cells from infected/treated mice to trigger efficient secondary responses. In these experiments, animals were challenged with FrCas^E^-infected splenocytes on days 56 and 116 post-infection (infected/non-treated and infected/treated mice) and analyzed 9 days later for both the expansion and the killing activity of this cell pool. No significant memory response was found in infected/non-treated animals (**Figure 3A**), although the presence of a weak antiviral CD8^+^ T-cell response below the sensitivity of the assay could not formally be excluded (see below). In contrast, robust responses were detected in infected/treated mice at both time points tested (**Figure 3A**) (compare to the response observed before challenge with FrCas^E^-infected cells in **Figures 2B and 2C**). Moreover, a strong *in vivo* cytolytic activity (50-80%) directed to splenocytes loaded with the GagL peptide was observed in all infected/treated mice. Importantly, 3 out of the 5 infected/non-treated mice showed a weak but detectable cytolytic activity (9-20%) while the other 2 showed a basal cytolytic activity similar to that found in control non-infected/non-treated animals (<2.76%) (**Figure 3B**). Thus, CD8^+^ memory T cells from infected/treated mice can develop functional secondary responses.

Lastly, we found that infected/treated mice maintained a functional anti-FrCas^E^ cellular response in the long-term in a challenge experiment conducted 8 months post-infection in 10 infected/treated mice. Nine of the 10 mice had normal hematocrits and no sign of splenomegaly for the duration of the experiment, whereas one of them (mouse 172) showed clinical signs of leukemia from month 6 post-infection onwards due to partial protection by 667. Notably, (i) all mice except mouse 172 revealed a memory cellular CD8^+^ T-cell response in both flow cytometry- and *in vivo* CTL assays (**Figure 3C and 3D**) and (ii) no (7/9 mice) or very few (2/9 with 0.006 and 0.16%) SICs were found in the 9 non-leukemic mice. Thus, a transient 667 treatment shortly after infection led to a functional long-lasting memory CTL response whereas infected/non treated mice only developed a weak primary CTL response. This is consistent with the fact that most individuals of the former group did not develop leukemia while all of the latter group did.

### The 667 mAb enhances the virus-specific CD8^+^ T-cell response

**Figure 4:**
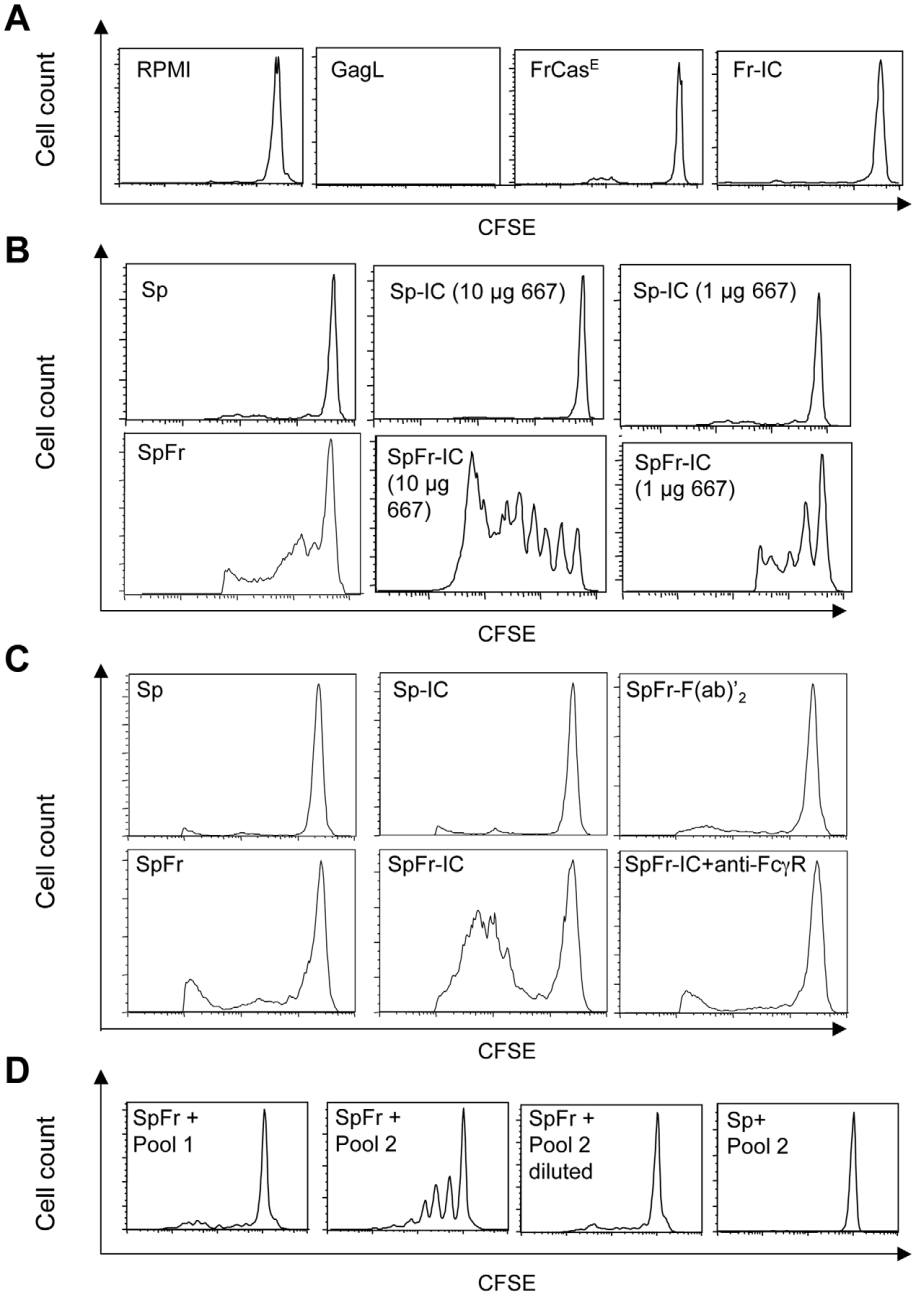
Stimulation of proliferation of GagL-TCR-TG CD8^+^ T cells by 667 and sera from infected/treated mice. BMDCs were prepared and exposed to various sources of antigen. The day after, they were co-cultured with CSFE-labeled CD8^+^ T cells prepared from lymph nodes from GagL-TCR-TG mice transgenic for a TCR recognizing the H2D^b^-restricted GagL peptide. Proliferation was assessed 5 days later by flow cytometry. (A) *Control experiments*. BMDCs were cultivated with the GagL peptide (used as positive control), FrCas^E^ and FrCas^E^ ICs made with 667 (Fr-IC). Culture medium (RPMI) served as a negative control. (B) *Stimulation of CD8^+^ T cells priming by FrCas^E^-infected splenocytes in the presence and in the absence of 667*. Non-infected (Sp) or infected splenocytes (SpFr) were incubated in the presence of 2 concentrations of 667 (Sp-IC and SpFr-IC, respectively). A typical experiment out of 3 independent ones with similar outcomes is presented. The statistical significance between the SpFr and SpFr-IC groups in these 3 experiments was established using the Student's t test (*P = 0,0003) (see **Figure S4**). (C) *Stimulation of CD8^+^ T cells priming by FrCas^E^-infected splenocytes complexed with either the 667 F(ab')_2_ fragment or the whole 667 mAb in the presence and in the absence of the FcγR-blocking antibody.* FrCas^E^-infected splenocytes (SpFr) were complexed with either 15 µg of the 667 F(ab')_2_ (SpFr- F(ab')_2_) or with 10 µg of the 667 mAb (SpFr-IC). SpFr-IC were co-cultured with BMDCs previously incubated (SpFr-IC + anti-FcγR), or not, with the FcγR-blocking antibody. Non-infected splenocytes incubated in the absence (Sp) or in the presence of 10 µg of 667 (Sp-IC) were used as control. (D) *Stimulation of CD8^+^ T cells priming by FrCas^E^-infected splenocytes in the presence of sera from infected/treated mice or infected/non-treated mice.* Pool 1 corresponds to sera from 4 infected/non-treated animals sacrificed on day 115 post-infection whereas Pool 2 corresponds to the sera of 4 infected/treated mice sacrificed on day 243. “Pool 2-diluted” corresponds to Pool 2 with anti-FrCas^E^ antibody concentration adjusted to that of Pool 1. Similar results were obtained in 2 independent experiments with individual serum samples from mice of each group.

**Figure 5:**
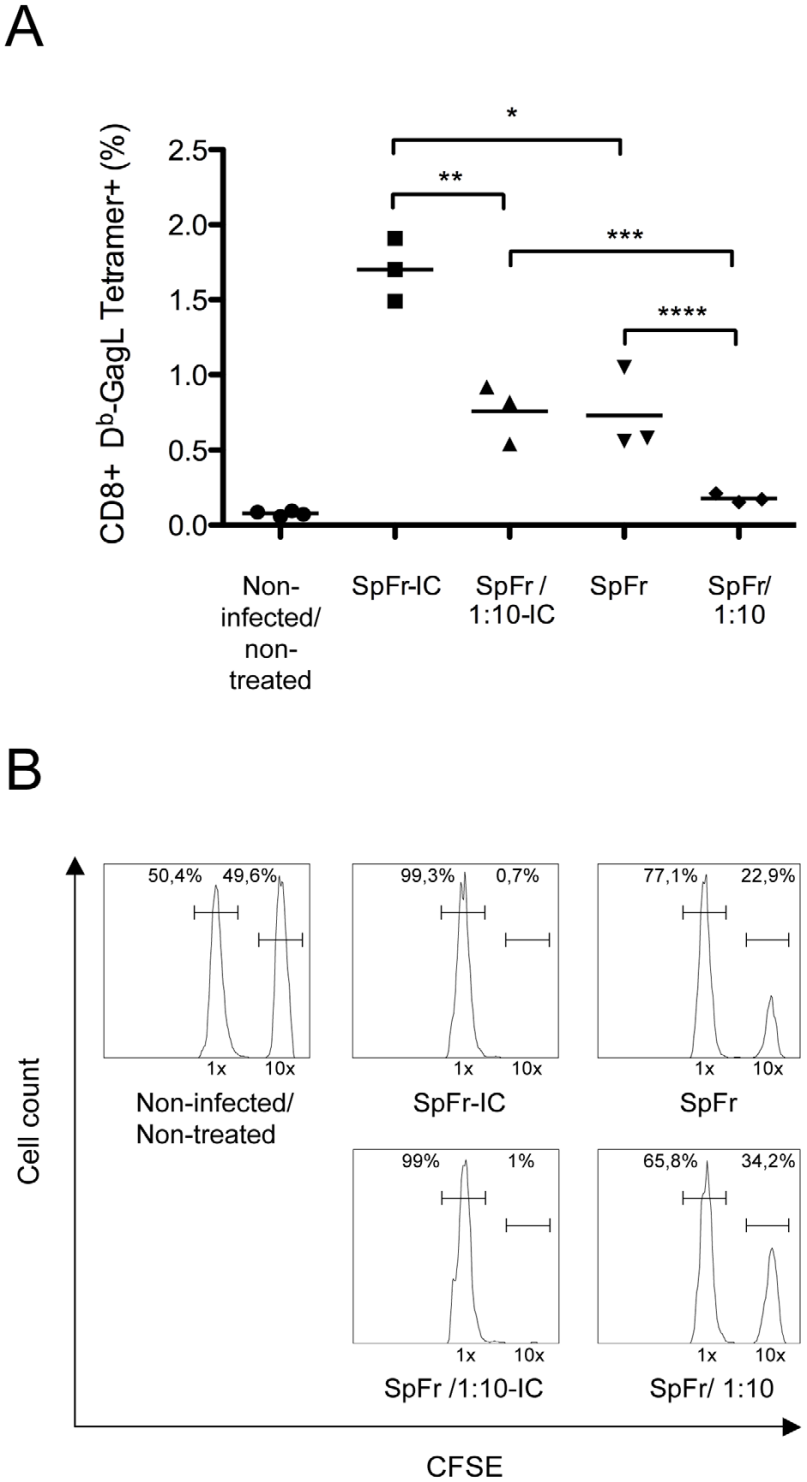
*In vivo* enhancement of CD8^+^ T-cell responses against FrCas^E^ by ICs. SpFr or SpFr-IC were administered i.v. to non-infected/non-treated mice. Four groups of mice (*n* = 3) where used: two of them were injected with either 2×10^6^ or 2×10^5^ FrCas^E^-infected splenocytes (SpFr and SpFr 1:10, respectively) in the absence of 667, and the other two were injected with the same amount of FrCas^E^-infected splenocytes complexed to 150 µg of 667 (SpFr-IC and SpFr 1:10-IC respectively). Nine days later, they were tested for the expansion of GagL-specific CD8^+^ T cells and for CTL activity *in vivo* as described in **Figure 3**. Non-infected/non-treated mice with no further treatment were used as controls *(A). Proliferation of GagL-specific CD8^+^ cells.* The statistical significance between groups was established using the Student's t test (*P = 0,0106; **P = 0,0049; ***P = 0,0071: ****P = 0,0352). (B) CTL activity against GagL-loaded splenocytes. A representative mouse of each group is presented.

To understand the enhanced cellular immunity mounted by infected/treated mice, we tested whether ICs formed by 667 and viral antigens during passive immunotherapy could prime the antiviral CTL response more efficiently than FrCas^E^ alone. To this aim, bone marrow-derived dendritic cells (BMDCs) were pulsed with (i) ICs made up of FrCas^E^ +667 (Fr-IC) or FrCas^E^-infected splenocytes +667 (SpFr-IC) or (ii) non-complexed FrCas^E^ or splenocytes simply infected with FrCas^E^ (SpFr). Their ability to stimulate the proliferation of CFSE-labeled CD8^+^ naive T cells from transgenic mice (GagL-TCR-TG) expressing a TCR specific for H2D^b^-restricted GagL [34] was then assessed *in vitro*. Unloaded- and GagL-loaded BMDCs were used as negative and positive controls, respectively. No GagL-TCR-TG CD8^+^ T-cell proliferation was observed in the presence of either FrCas^E^ alone or FrCas^E^ complexed with 667 (Fr-IC) (**Figure 4A**). This was not surprising as GagL is derived from gPr80^Gag^, which is not incorporated into virions [33]. In contrast, both infected splenocytes (SpFr) and infected splenocytes complexed with the 667 mAb (SpFr-IC) stimulated proliferation (**Figure 4B**) with a stronger effect for SpFr-IC. This showed that cellular ICs were more efficient than simply infected cells at stimulating the proliferation of FrCas^E^-infected cell-specific CTLs by BMDCs (**Figure 4B** **and Figure S4**). We next addressed whether this increased T cells proliferation was dependent on FcγRs expressed by DCs. This turned out to be the case as proliferation of GagL-specific CD8^+^ T cells was decreased (i) when the experiment was conducted in the presence of a FcγR-blocking mAb (2.4G2 mAb; [35]) or when (ii) cellular ICs were made with a F(ab)'_2_ fragment of 667 (SpFr-F(ab)'_2_) instead of the whole 667 mAb (**Figure 4C**). Importantly, we extended these observations to the *in vivo* situation. Two different amounts of FrCas^E^-infected splenocytes, either complexed with 667 (SpFr-IC and SpFr/1:10-IC, respectively) or non-complexed (SpFr and SpFr/1:10, respectively), were administered i.v. to mice and CTL responses were analyzed 9 days later. ICs were more efficient at both stimulating proliferation of GagL-specific CD8^+^ T cells (**Figure 5A** and **Figure S5**) and enhancing CTL responses against infected cells (**Figure 5B** **and Figure S5**) than non antibody-complexed infected splenocytes regardless of the amount of cells used. These results indicated that 667-involving cellular ICs enhance CD8^+^ T-cell responses *in vivo.*

### 667 enhances uptake of infected cells and induces more efficient activation of DCs

**Figure 6:**
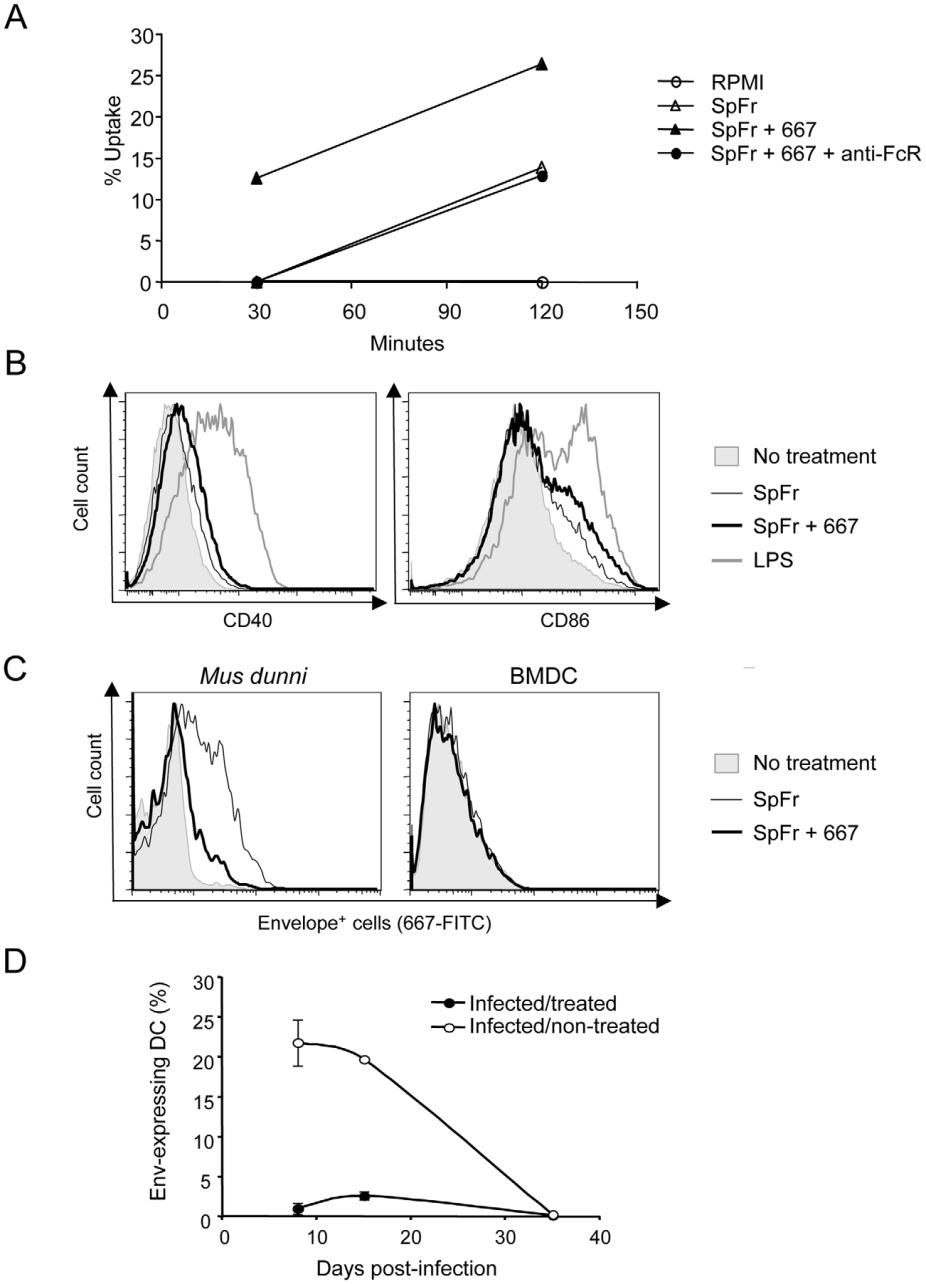
Influence of FrCas^E^-infected cell ICs on BMDCs. (A) *Uptake of SpFr and SpFr-IC by DCs.* PKH26-labelled SpFr or -SpFr-IC were given to BMDCs and uptake was quantified by flow cytometry in the presence, or in the absence, of the 2.4G2 FcγR-blocking mAb. One experiment out of 3 with similar outcomes is presented. (B) *BMDCs activation*. BMDCs were incubated for 24 hours in the presence of SpFr, SpFr-IC or LPS before flow cytometry assay of CD40 and CD86. One representative experiment out of 3 independent with similar outcomes is presented. (C) *Assessment of productive* in vitro *BMDC infection.* BMDC were co-cultured with SpFr or SpFr-IC for 48 hours before flow cytometry assay of MHC-II^+^Env^+^ cells. Infection-sensitive *Mus dunni* fibroblasts were used as positive controls in parallel experiments. (D). *Assay of Env-expressing DCs* in vivo. MHC-II^+^Env^+^ splenocytes from infected/treated- and infected/non-treated mice were assayed by flow cytometry at days 8, 15 and 35 post-infection.

Antigens in the form of ICs have already been reported to be, not only more efficiently taken up by DCs, but also better activators of these cells than free antigens [13], [36]. As a first step to explain the stronger anti-FrCas^E^ CTL responses presented in **Figures 4** **and** **5**, we therefore reasoned that DCs could take up SpFr-IC more efficiently than SpFr, which would lead to their stronger activation. Uptake by DCs was investigated as follows. First, SpFr were stained with the membrane-labelling dye PKH26 and complexed, or not, with 667. Then, they were given to DCs in the presence or in the absence of the FcγR-blocking 2.4G2 mAb for various periods of time before flow cytometry quantification of the PKH26 fluorescence present in DCs. The data of **Figure 6A** show that SpFr-IC were taken up faster and more efficiently than SpFr and that differential uptake efficiency was dependent on FcγRs. These data were consistent with those of **Figure 4C** showing that the FcγR-blocking mAb inhibits GagL-TCR-TG CD8^+^ T-cell proliferation by SpFr-IC-activated BMDCs. We then quantified the abundances of the CD40 and CD86 co-stimulatory molecules at the surface of BMDCs cultured in the presence of SpFr or SpFr-IC, as these markers are classically used to monitor DC activation. Both of them were slightly but reproducibly more expressed in the latter case (**Figure 6B**), indicating better activation of BMDCs by SpFr-IC.

As a significant fraction of DCs can express retroviral proteins in mice infected with MuLVs [37], we also considered that cellular ICs may have favored DC-, or DC precursor, infection by FrCas^E^ and, hence, led to the generation of DCs with altered properties possibly influencing CTL responses in infected/treated mice. In a first step, we asked whether uptake of SpFr, complexed or not with 667, could be followed by productive infection of DCs. This was achieved by assaying Env expression 48 hours after giving SpFr or SpFr-IC to BMDCs. As positive controls, SpFr or SpFr-IC were given to infection-sensitive *Mus dunni* fibroblasts in parallel experiments. Thirty and 6% of *Mus dunni* cells turned out to be positive for Env after infection in the presence of SpFr or SpFr-IC, respectively, lower infection in the latter case being consistent with a neutralization action of 667 (**Figure 6C**). In contrast, BMDCs were negative whatever the condition tested (**Figure 6C**). This was not surprising as BMDCs are essentially post-mitotic cells under the experimental conditions used and MuLVs require mitosis for proviral integration and subsequent viral function expression. As proliferating FcγR-expressing precursors of DCs might possibly be infected *in vivo* and lead to the generation of DCs with altered properties due to the expression of retroviral proteins, we also monitored Env positivity of splenic DCs in both infected/non-treated and infected/treated mice in the post-infection period (**Figure 6D**). 20% of splenic DCs were Env-positive in infected/non-treated mice on both days 8 and 15 post-infection. In contrast, only 1% and 3% of their counterparts were positive in infected/treated mice at the same time points and no Env-positive cell was detected at a later time (day 35). Thus, rather than enhancing the number of DCs expressing viral Env *in vivo*, the treatment by 667 diminishes it, presumably because reduced viral spread in infected/treated mice limits infection of DC precursors. In conclusion, stronger stimulation of anti-FrCas^E^ CTL responses by cellular ICs correlates with more efficient uptake by DCs followed by more efficient activation but is unlikely to involve infection of DCs or DC precursors.

### Enhancement of CD8^+^ T-cell response against infected cells by endogenous anti-FrCas^E^ antibodies generated in infected/treated mice

As infected/treated mice developed a long-lasting CD8^+^ T-cell response correlating with high titers of anti-FrCas^E^ antibodies, we next studied whether the humoral response in infected/treated mice could contribute to the long-lasting maintenance of the CTL immunity via a mechanism similar to that of 667. To address this, pooled sera from either 4 infected/non-treated mice (pool 1) sacrificed on day 115 post-infection or 4 infected/treated mice (pool 2) sacrificed on day 243 were used to form ICs with infected splenocytes in the T-cell proliferation assay described above. Because anti-FrCas^E^ antibody titers were higher in infected/treated mice (120 vs. 70 µg/ml), we compared the CD8^+^ T-cell priming efficiencies of the 2 serum pools at both identical dilutions and at identical anti-FrCas^E^ antibody concentrations. A GagL-TCR-TG CD8^+^ T cell proliferation was detected in the presence of sera from infected/treated mice (pool 2) but not in the presence of sera from infected/non-treated mice (pool 1) when used at identical dilutions (**Figure 4D**). In contrast, no proliferation was seen with either pool of sera when used at identical anti-FrCas^E^ concentrations (pool 1 versus pool 2-diluted) (**Figure 4D**). This indicated that endogenous antibodies from infected/treated mice promoted CD8^+^ T cell proliferation through the formation of ICs with FrCas^E^-infected cells. However, this effect was dose-dependent, which may explain why the CD8^+^ T-cell response was weaker in infected/non-treated mice than in infected/treated ones as the former displayed lower titers of anti-FrCas^E^ antibodies. These results suggest that the enhanced endogenous humoral response induced in infected/treated mice plays an important role in the maintenance of the cellular response after the administered 667 mAb has disappeared.

### 667 and anti-FrCas^E^ antibodies endogenously generated in infected/treated mice show cytolytic activity *in vivo*

**Figure 7:**
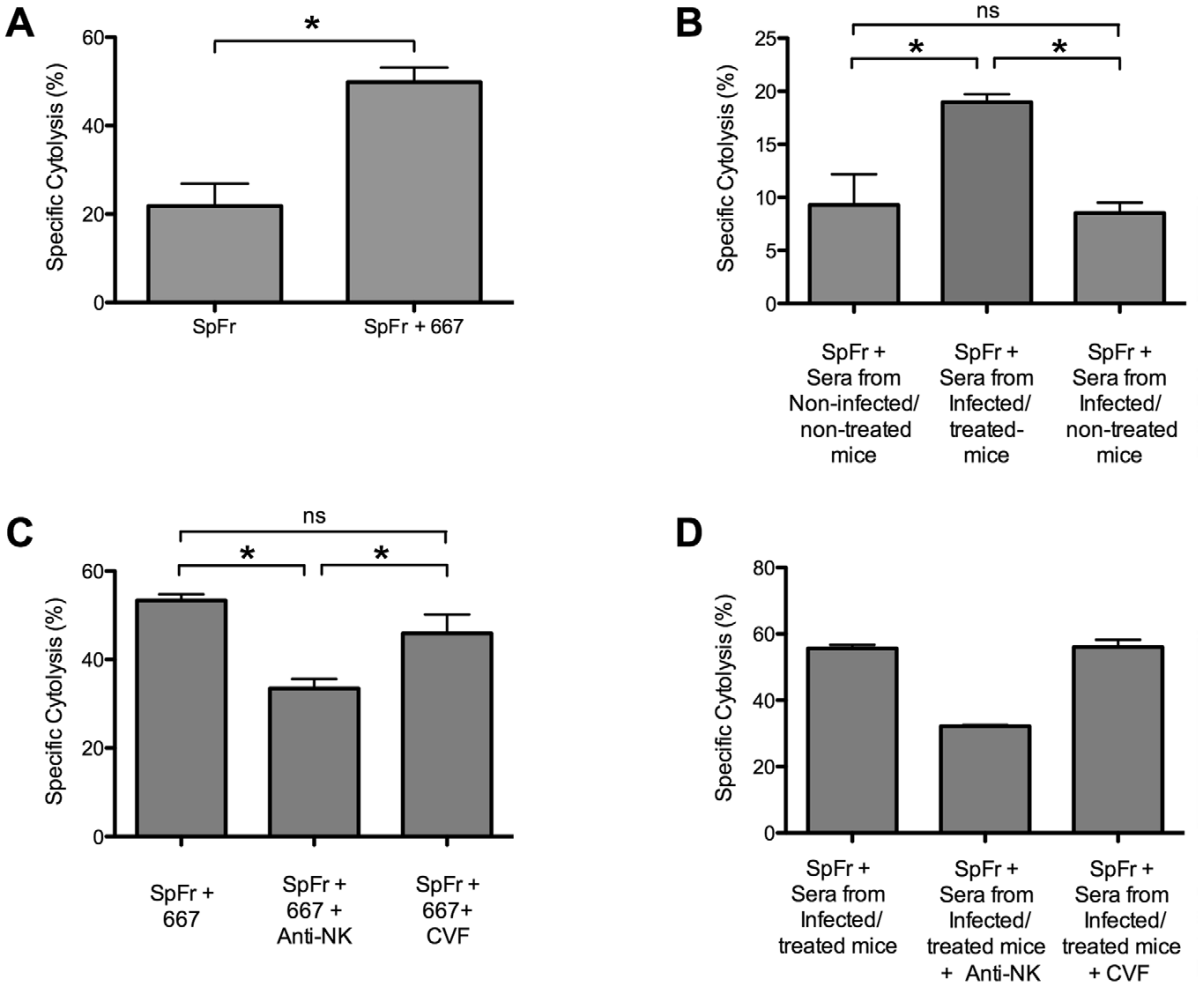
Cytolytic activity against FrCas^E^-infected cells of 667 and of anti-FrCas^E^ antibodies endogenously produced in infected/treated mice. *In vivo* cytolysis activities were assayed after administration of CFSE-labeled FrCas^E^-infected splenocytes (SpFr) followed by administration of antibodies from various sources and cytometry analysis after 5 or 24 hours. (A) *Cytolytic activity of 667*. SpFr were administered i.v. and, then, 667 or PBS i.p. Values are the average ± SEM of experiments conducted with 4 mice per condition. The statistical significance was established using the Student's t test (*P = 0,0007). (B) *Cytolytic activity of sera from infected/treated- and infected/non-treated mice*. After administration of SpFr, three groups of 4 mice each were treated with equivalent volumes (175 µl) of pools of sera from 2 non-infected/non-treated-, 2 infected/non-treated- and 2 infected/treated mice. Values are the average ± SEM of experiments conducted with 4 mice per condition. Statistical significance of sera from infected/treated group versus the other two groups was determined by a non-parametric one-way ANOVA test with Dunn's multiple comparison post-test. *P<0,05. (C) *Cytolytic activity of 667 in mice treated with either anti-NK antibodies or cobra venom factor*. SpFr mixed with 667, or PBS for control, were administered i.v. to mice treated or not with either anti-NK antibodies or the cobra venom factor as described in the Methods section. The values are the average ± SEM of experiments conducted with 7 mice per condition. The statistical significance of the data obtained with sera from the infected/treated group versus those obtained in the other two groups was established using a non-parametric one-way ANOVA test with Dunn's multiple comparison post-test. (*P<0,05). (D) *Cytolytic activity of sera from infected/treated in mice treated with anti-NK antibodies or cobra venom factor*. SpFr mixed to 175 µl of pools of sera from 4 infected/treated were administered i.v. to mice treated or not with either anti-NK antibodies or the cobra venom factor, as described in the Methods section. The values are the average ± SEM of experiments conducted with 2 mice per condition. Statistical significance of sera from infected/treated group versus the other two groups was not assessed due to the reduced number of mice per condition.

Finally, it was important to assess whether 667 and anti-FrCas^E^ antibodies generated endogenously in infected/treated mice could lyse infected cells *in vivo*. To this end, we injected i.v. a mixture of non-infected- and FrCas^E^-infected splenocytes in 2 month-old naive mice. This was followed by i.p. administration of either (i) 667 or control PBS or (ii) pools of 2 sera from 3 month-old non-infected/non-treated, infected/non-treated or infected/treated mice. In the case of 667, the ratio of the two cell types was assayed by flow cytometry of circulating blood cells 5 hours later. In addition to a spontaneous basal cell lysis activity, we observed a rapid cytolytic activity against FrCas^E^-infected cells due to 667 (**Figure 7A**). For the experiments with the pools of sera, comparable volumes of sera were injected to the mice and cytolysis was assayed 24 hours later (**Figure 7B**). Cytolysis activity of sera from infected/non-treated mice was comparable to spontaneous cytolysis in control naive mice whereas that of sera from infected/treated animals was much stronger. Thus, both 667 and anti-FrCas^E^ antibodies contained in sera from infected/treated mice can lyse FrCas^E^-infected cells *in vivo*. However, the *in vivo* cytolysis of infected cells observed by 667 administration was higher than the one observed by sera from infected/treated mice. This was most probably due to the fact that volume of pooled sera used contained about 100 µg of anti-FrCas^E^ antibodies while 200 µg of 667 mAb were administrated, suggesting that anti-viral antibody concentration might be a critical factor to induce efficient cell lyses of infected cells.

As a last step, we addressed whether antibody-mediated lysis of infected cells was dependent on NK cells and/or complement. To this aim, new *in vivo* cytolysis experiments were conducted after either having depleted NK cells by administration of an anti-asialo GM1 antibody or inactivated complement activity through injection of the cobra venom factor. To increase the efficiency of our assay, the mixture of non-infected- and FrCas^E^-infected splenocytes was, this time, pre-incubated with either the 667 mAb or anti-FrCas^E^ antibodies from infected/treated mice serum prior to administration. In the latter case, we also increased the quantity of anti-FrCas^E^ antibodies administrated to mice by pooling sera from mice with high serum anti-viral antibody concentrations. NK cell depletion resulted in a dramatic decrease of infected-cell lysis by both the 667 antibody (**Figure 7C**) and the sera from infected/treated mice (**Figure 7D**) whereas inactivation of the complement system had no detectable effect (**Figure 7C and 7D**). This pointed to a role for ADCC in infected lysis by both 667 and the endogenous antiviral humoral response of infected/treated mice.

## Discussion

Our data point to an immunomodulatory effect of the 667 mAb, which permits FrCas^E^-infected mice to mount a long-lasting protective immunity comprised of both a strong neutralizing humoral response and a robust CTL contribution that subtly interact to protect individuals from virally-induced diseases in the long-term. This “vaccine-like” effect, which lasts long after the termination of the treatment, contrasts with the non-protective immune response in infected/non-treated controls. If applicable to other persistent viral infections, our observation will have important therapeutical consequences for mAb-based immunotherapies. One of our most notable findings is that, not only the generation of an efficient primary CTL response, but also the activation of the long-lasting antiviral memory CD8^+^ T cells involves the formation of ICs between antibodies and infected cells. Therefore, our data strongly suggest that, antibodies targeting proteins present, not just on virions, but also on the surface of infected cells should have a crucial advantage for efficient treatment of virally-induced diseases in the context of passive immunotherapies.

### How might protective antiviral immunity be initiated in 667-treated, FrCas^E^-infected mice?

Several non-exclusive mechanisms may explain the long-term consequences of the 667 treatment on endogenous antiviral immunity. Firstly, our data show a strong antiviral effect of this mAb on both plasma viremia and SICs before an adaptive immune response could emerge. This suggests that an immediate effect of 667 might be the blunting of viral propagation that would indirectly provide time for the immune system to mount a protective antiviral immunity before being overwhelmed. Our data also strongly suggest that the potent effect of 667 on the viral load is due to, not only its neutralizing activity, but also to its *in vivo* cytolytic activity against FrCas^E^-infected cells mainly through ADCC-mediated mechanisms. Secondly, due to 667 ability to enhance the priming of CD8^+^ T cells directed to FrCas^E^-infected cells, it is unlikely that limiting viral propagation is the sole mechanism whereby this mAb impacts on endogenous humoral and cellular anti-FrCas^E^ responses. Rather, direct immunomodulatory mechanisms involving the formation of ICs, not only with viral particles but also with infected cells exposing Env at their surface, seem essential. Indeed, IgGs have already been shown to act as natural adjuvants in a process called antibody feedback regulation to enhance immune responses against antigens they are specific for in various settings, including infection by pathogens [38]. One major mechanism whereby administered antibodies operate is interactions of ICs with the FcγRs expressed by various immune cell types, including DCs. Such interactions lead to both enhanced humoral- [39]–[42] and stronger CD4^+^ and CD8^+^ T-cell responses due to more efficient DC activation/maturation characterized by better generation and presentation of antigenic peptides [14], [36], [43]. It is however important to stress that the potential therapeutic effect of IC-DC interactions has, thus far, principally been studied in the context of antitumoral immunotherapies using *in vitro* techniques [24], [44] or *in vivo* approaches that mainly rely on the simplified OVA model system and/or the grafting of *in vitro* IC-loaded DCs [14], [36], [45], but it has never been addressed in the context of real persistent/lethal viral infections. Therefore, our work demonstrates for the first time that cellular ICs formed during a mAb-based passive immunotherapy can be crucial for inducing a long lasting protective antiviral immunity. It also shows that interactions of such IC with FcγRs expressed on DCs are important in the induction of enhanced CD8^+^ T-cell responses. Supporting the idea that our findings may also apply to infections by persistent viruses other than FrCas^E^, (i) enhanced humoral responses have been described upon use of ICs in therapeutical vaccination-based treatments of HBV infection [46] and (ii) *in vitro* stimulation of peripheral blood mononuclear cells from SIV-infected macaques with ICs made up of p55^gag^ + anti-p55^gag^ IgGs led to more efficient stimulation of precursor CTLs against SIV-infected cells than stimulation by p55^gag^ alone [47]. However, no data concerning protection from disease was reported in the latter study.

How does 667 induce long-lasting protective humoral- and cellular anti-FrCas^E^ immunities? IgG responses were significantly different in intensity and antiviral activity between infected/treated and infected/non-treated mice (**Figure 1C**), suggesting an immunomodulatory effect of 667 affecting both the magnitude and the quality of the anti-FrCas^E^ humoral response. Importantly, an enhanced antibody response following short passive immunotherapy is unlikely to be exclusive to our model as intensive treatment of juvenile SIV-infected macaques with anti-SIV hyperimmune serum Igs accelerated the appearance of neutralizing antibodies, which correlated with delayed onset of the disease in some animals [48]. As ICs play an important role in the development of antibody responses via different modes of action [13], [40]–[42], further work will be needed to address how 667-containing ICs can induce enhanced humoral immunity. As for the cellular response, the most likely scenario for enhanced CTL response in infected/treated mice is that 667 would initially target FrCas^E^-infected cells and lyse them by ADCC. The resulting cellular immune complexes would then be efficiently taken up by DCs, owing to interactions with FcγRs, for more efficient stimulation of effector cells of the immune system. Several of our observations support this scenario: (i) uptake of FrCas^E^-infected cells by DCs is increased by 667 and this increase is inhibitable by a FcγR-specific antibody (**Figure 6A**), (ii) DCs are more efficiently activated by SpFr-IC than by SpFr, at least as assayed by CD40, and CD86 expression (**Figure 6B**), (iii) 667-complexed FrCas^E^-infected splenocytes are more efficient at inducing anti-FrCas^E^ CD8^+^ T-cell responses than non-mAb-complexed cells both *in vitro* (**Figure 4B**) and *in vivo* (**Figure 5**) and (iv) such enhanced CD8^+^ T-cell responses are dependent on IC interactions with FcγRs expressed on DCs (**Figure 4C**). One challenge is now to elucidate the intimate cellular and molecular mechanisms whereby cellular IC-activated DCs can induce stronger immune responses than simply infected cells. Obvious non exclusive processes that need to be assessed include (i) the facilitation of DC migration to lymphoid organs, (ii) the increased production of proinflammatory cytokines production and/or decreased production of immunosuppressive ones or (iii) the better processing of cellular antigens and/or presentation to effector cells as reported in other systems [13], [36], [43], [45], [49]. Moreover, one will have to take into consideration that, *in vivo*, cellular IC-activated DCs may interact with, not only CTLs, but also other effector cells such as CD4^+^ lymphocytes. Whatever the mechanisms involved, our work importantly shows that uptake of cellular ICs allows the presentation of the full viral antigenic repertoire, including determinants not incorporated into virions but expressed only in infected cells (as shown here by the presence of CD8^+^ T cells specific for GagL epitope from gPr80^gag^). This is probably crucial for rejection of infected/leukemogenic cells by CTLs, as suggested by efficient lysis of GagL-loaded splenocytes administered to infected/treated mice (**Figures 3B, 3D** **and** **5B**). Supporting this idea, the coating of apoptotic tumor cells with antibodies enhances antitumoral immune responses in various settings [13], [24], [44], [50]. Finally, as 667 is able to target infected cells *in vivo*, it is plausible that 667-induced cell lysis through ADCC-mediated mechanisms may create an inflammatory environment favoring uptake, processing and presentation of viral antigenic determinants by DCs to T cells as reported in the case of lytic anti-tumor mAb [**51**], [**52**]. In conclusion, 667/infected cells ICs appear to have a two-fold effect: they enhance quantitatively and qualitatively viral antigen presentation, which induces potent CTL responses directed to infected cells.

### How might protective antiviral immunity be maintained in infected/treated mice after the disappearance of 667?

Our data strongly suggest that long-term protective antiviral immunity in infected/treated mice relies on a complex relationship between humoral and cellular anti-FrCas^E^ immunities following the primary response. In this respect, a first question is why the humoral anti-FrCas^E^ response lasts so long and is so high in infected/treated mice as compared to infected/non-treated ones? Two lines of evidence argue that this is likely due to persistent stimulation by residual infected cells with an intensity positively correlated to the number of these cells as (i) the humoral response begins to decrease by the moment SICs become undetectable (**Figure 1B–C**) and (ii) individual animal analysis at month 8 post-infection/treatment (unpublished data) shows that anti-FrCas^E^ antibody titers are higher in mice with residual SICs compared to those with undetectable SICs (the virus probably not being eradicated in the latter mice). A second important issue is why the CTL response is maintained at a low level in infected/treated animals except under conditions of challenge? As endogenous anti-FrCas^E^ antibodies can lyse FrCas^E^-infected cells, we hypothesize that, after establishment of persistent infection, the CTL pool is weakly or no longer solicited, as suggested by the low levels of effector-like memory-Tcells detected in unchallenged infected/treated mice (**Figure 2**). This would avoid exhaustion by overstimulation and, consequently, preserve the possibility of strong anamnestic responses in case of virus reactivation as described in various infection systems [53], [54]. Supporting this idea, virus-specific antibodies have already been shown essential for long-term maintenance of the memory CTL response upon infection of mice by the lymphocytic choriomeningitis virus (LCMV) [27], [53]. Finally, it is also important to consider that the humoral response might facilitate the activation of memory CTLs in case of viral reactivation/reinfection (mimicked here by a challenge with infected cells), as we show that anti-FrCas^E^ antibodies, through the formation of ICs, can enhance the proliferation of CTLs specific for infected cells (**Figure 4D**). Strengthening this idea, elicitation of such specific CTL responses by adequate levels of antibodies directed against tumor antigens has recently been reported in therapeutical vaccination experiments against cancer cells [55]. Thus, our work shows that the endogenous immune response induced by the immunotherapy can take over the administered mAb to contain viral propagation long after the end of the treatment. It also suggests that this antiviral immunity is capable of self-maintenance through a complex balance between the humoral and cellular arms of the immune response with a critical role for ICs.

### MAb-based immunotherapies and treatment of human chronic infections

Most available therapies against severe chronic viral infections are based on the use of pharmacological drugs and/or interferon [56], [57]. Their aim is to inhibit viral spread/replication via various mechanisms but not to enhance the antiviral immune response of treated patients. In certain cases, they can even lead to “immune amnesia” due to the dramatic decrease of viral antigens, as it has been reported in the case of HIV [58]–[60]. Therefore, these therapeutical approaches clearly depart from ours, which shows that, in addition to its direct antiviral effect, a passive immunotherapy can also favor the mounting of a protective antiviral immunity. One important mechanistic explanation for the different immune outcomes between the two types of treatments is that antiviral drugs do not enhance viral antigen presentation by APCs whereas antiviral mAbs can through an opsonized antigen-based process, as shown here. Consequently, our work identifies a thus far underappreciated immunomodulatory property of antiviral mAbs and underlines a possibly major difference with other antiviral drugs.

Our experiments were conducted in the context of early post-exposure therapy of young animals with still-developing immune systems. Moreover, the neutralizing mAb was administered rapidly after infection. It is therefore possible that the application of such passive immunotherapy-stimulated antiviral immunity approaches may be limited to cases of suspicion of infection in young individuals by life-threatening viruses such as HCV or HIV. An important challenge will now be to determine whether passive mAb-based immunotherapies can also influence the immune response of chronically infected adult mice. Whatever the answer to this question, the fact that our experiments were conducted with young animals still in the period of immune system maturation raises the possibility that a possible first application of this effect might be the treatment of mother-to-child transmission of HIV for two reasons: the need of novel therapies because of spontaneous resistances of certain patients to current chemotherapies and the availability of neutralizing mAbs against this virus (see introduction). During the first 12–48 months, maternally-transmitted neutralizing antibodies from immunized/vaccinated mothers protect babies and infants from various acute infections and may turn incoming viruses into effective vaccines in a process called “natural vaccination” [61]. However, mothers who are HIV carriers usually do not produce neutralizing antibodies and can transmit the virus to babies at birth and natural vaccination cannot take place. An important question would therefore be whether the administration of exogenous anti-HIV mAbs to perinatally infected babies could reproduce the “natural vaccination” observed in the case of other viral infections. The fact that, as already cited, the treatment of juvenile SIV-infected macaques with anti-SIV hyperimmune serum Igs accelerated the appearance of neutralizing antibodies and was followed by delayed onset of the disease in certain monkeys [48] provides a support to this idea.

## Methods

### Ethics statement

All experimental procedures performed in this study are in accordance with the local animal facility “ComEth” Institutional Review Board guidelines, and have been approved by the local animal facility “ComEth” Institutional Review Board under the supervision of the French LR Regional CEEA ethic committee on animal experimentation (Chairman: Pr M Michel, Montpellier).

### Viral stocks

FrCas^E^ viral stocks were produced as described in [17].

### Monoclonal antibody and F(ab')_2_ fragments production

The anti-MuLV Env mouse 667 (IgG2a) and 678 (IgG1) mAbs [21] were purified as previously described [20]. The 667 F(ab')_2_ was prepared as follows: 667, at concentration of 3 mg/ml in 20 mM sodium acetate (pH 4.0) was mixed with pepsin at concentration of 1 mg/ml in 20 mM sodium acetate (pH 4.0). The mixture was incubated for 7 hours at 37°C. The undigested mAb was removed by protein A column chromatography. The unbound fraction was collected as the F(ab′)_2_ and purity was checked by sodium dodecyl sulfate-polyacrylamide gel electrophoresis analysis. The activity of the F(ab′)_2_ was shown identical to that of intact 667 by an *in vitro* plaque reduction neutralization assay. The control anti-β-2,6-fructosan IgG2a mAb is from Sigma. The FcγR-blocking 2.4G2 mAb [35], which recognizes both CD16 and CD32, was purchased from BioXcell and the anti-asialo GM1 mAb used for NK cells depletion was from Wako.

### Virus titers and *in vitro* neutralization activity assays

Viral titers were determined using a focal immunofluorescence assay (FIA) [62] as previously described [18], [20], [22]. Virus neutralization activity of mouse sera, was assayed as previously described [18]. Briefly, 4×10^2^ FrCas^E^ ffu (focus-forming units) were diluted in a 1∶1 ratio with serum samples and incubated at 37°C for 1 hour. Mixes were used to infect target cells overnight. Cells were allowed to reach confluence, at which time ffu were scored by FIA.

### Viral infection and follow-up of mice

Inbred 129/Sv/Ev mice (H-2D^b^ haplotype) were used in this study. Eight day-old mice were infected i.p. with 100 µl of a virus suspension containing 5×10^5^ ffu/ml and treated or not with 30 µg of 667 or the control anti-β-2,6-fructosan mAb one hour post-infection and on days 2 and 5 post-infection by i.p. administration. Mice were examined at regular intervals for clinical signs of erythroleukemia (spleen swelling and reduction in hematocrits). Mice were sacrificed when their hematocrits reached 35% to avoid unnecessary pain, as they would die within the next days. Spleens were weighted either after death or on the day of sacrifice. Viremia was assayed from sera as previously described [17], [18].

### ELISA of anti-FrCas^E^ antibodies

The 667 mAb and serum anti-FrCas^E^ antibodies were assayed as described previously [17], [18]. The 667 and 678 mAbs were used as standards for quantifications of anti-FrCas^E^ IgG2a and IgG1 antibodies, respectively.

### Spleen infectious center (SIC) assays

Serial dilutions of single-cell suspensions from FrCas^E^-infected mouse spleens prepared as in ref. [22] were plated on 25% confluent indicator *Mus dunni* cell cultures. After 3 days of co-culture, ffu were score by FIA [see 18], [62]

### Stimulation of memory CTL responses

Mice were injected i.v. with 0.6×10^6^ FrCas^E^-infected splenocytes resuspended in 100 µl of PBS.

### Assay of CD8^+^ T cells specific for FrCas^E^-infected cells by flow cytometry

5×10^5^ splenocytes were stained with both an anti-CD8-APC- (Becton Dickinson) and a PE-labeled MHC class I H-2D^b^ tetramer (Beckman Coulter) specific for the Friend Virus GagL peptide (D^b^-GagL tetramers) [32], [63] for 15 min at room temperature in PBA (PBS containing 2% FCS and 0.01% sodium azide). Cells were analyzed by flow cytometry after paraformaldehyde fixation. Alternatively, CD3^+^ cells were isolated from spleen by a negative selection kit (Invitrogen Dynal) and stained with the D^b^-GagL tetramers and anti-CD8, anti-CD62L (Becton Dickinson), anti-CD44, anti-CD127 and anti-CD25 mAbs (eBioscience) and analyzed by flow cytometry. IFN-γ production in CD3^+^ cells cultured in the presence of PMA-ionomycin and brefeldin A was measured by using the intracellular staining kit (Cytofix/Cytoperm Fixation/Permeabilization, Becton Dickinson) according to the supplier's protocol.

### *In vivo* cytotoxicity assays

Experiments were conducted as described in [22]. Briefly, red blood cell-free splenocytes from naive mice were either pulsed with the GagL peptide [33] or kept unloaded to use them as control splenocytes for quantification of spontaneous cell death in adoptive transfer experiments. GagL-loaded- and control splenocytes were labelled by incubation in either 5 µM CFSE (CFSE^high^ cells)- or 0.5 µM CFSE (CFSE^low^ cells)-containing PBS, respectively. Labeled cells were mixed in a 1∶1 ratio and 10^7^ cells of the mix were injected i.v. in each recipient 8 days after viral challenge. 16 hours later, mice were sacrificed for flow cytometry quantification of splenic CFSE^low^- and CFSE^high^ cells. The CTL activity against GagL-loaded splenocytes was calculated from the ratio of CFSE^high^/CFSE^low^ cells.

### Preparation and characterization of BMDCs and phenotyping of retroviral expression in BMDCs and spleen DCs

Functional studies were performed with DCs obtained from bone marrow precursors as described in ref. [64]. B- and T lymphocytes and macrophages were eliminated from bone marrow cells, which were cultured at 37°C for 7 days in 5% FCS-containing RPMI medium complemented with GM-CSF and IL-4 (200 U/ml each), 2 mM glutamine, 1% non-essential amino acids, 50 µM β-mercapto-ethanol and 1 mM sodium pyruvate in the presence of 5% CO_2_. At that time, they were used as immature DCs referred here to as BMDCs. They were 80–90% pure, as assayed by flow cytometry for cells doubly positive for MHC class II and CD11c. BMDC activation was assessed by flow cytometry using mAbs directed to MHC-II (eBioscience), CD11c, CD40, and CD86 (Becton Dickinson) after 24 h of stimulation by SpFr, SpFr-IC or LPS (InVivoGen; 1 µg/ml). Retroviral Env expression on MHC-II^+^CD11c^+^ BMDCs, as well as on control *Mus dunni* cells, was also assayed by flow cytometry using the FITC-labeled 667 mAb after 48 hours of co-culture with SpFr or SpFr-IC. Co-expression of Env, CD11c and MHC-II on splenic DCs was assessed by flow cytometry 8, 15 and 35 post-infection after preparation of splenocytes from sacrificed mice.

### Assay of CD8^+^ T cells priming activity

ICs were formed by incubating 5×10^4^ FrCas^E^-infected splenocytes (SpFr) with either (i) 10 µg of the purified 667 mAb (SpFr-IC) or (ii) 15 µg of the 667 F(ab')_2_ fragment (SpFr- F(ab')_2_) or (iii) sera containing anti-FrCas^E^ antibodies (SpFr-serum). Non-immune sera were taken as controls in the latter case. 5×10^5^ BMDCs were co-cultured overnight with SpFr or SpFr-IC in the presence or in the absence of the 2.4G2 FcγR-blocking mAb, or with SpFr-F(ab')_2_ or SpFr-serum. After 2 washes in PBS, DCs were cultured with CD8^+^ T cells purified from lymph nodes of GagL-TCR-TG transgenic mice [34] (60% of CD8^+^ cells, of which 98% expressed the GagL-specific TCR, and labeled in the presence of 5 µM CFSE). Cell proliferation was followed up by flow cytometry analysis of CFSE- and CD8-positive cells 5 days later. Non-infected splenocytes were used as controls in these experiments. For *in vivo* experiments, 2×10^5^ or 2×10^6^ FrCas^E^-infected splenocytes (SpFr) were incubated or not with 150 µg of the purified 667 mAb and administered i.v. into naive mice to assess GagL-specific CD8^+^ T-cell expansion potential and cytotoxic activity as described above.

### Assay of *in vivo* anti-FrCas^E^ antibody cytolysis activity

The assay used is derived from ref. [65]. Non-infected and FrCas^E^-infected splenocytes were labeled with 0.5 µM (Sp CFSE^low^) and 5 µM (SpFr CFSE^high^) CSFE, respectively. They were then mixed in a 1∶1 ratio and administered i.v. to mice, which, afterwards, received either 200 µg of 667 or pools of sera containing anti-FrCas^E^ antibodies by i.p. administration. Alternatively, a mixture of non-infected and FrCas^E^-infected splenocytes at 1∶1 ratio was pre-incubated with either 667 or a pool of sera containing anti-FrCas^E^ antibodies from infected/treated mice prior to i.v. administration. The ratio of CFSE^low^/CFSE^high^ cells was assayed by flow cytometry 5 and 24 hours later, respectively. To assess the contribution of NK cells to antibody-mediated cytolysis, 50 µl of the anti-asialo GM1 anti-NK antibody was administrated i.v. to mice on days 6, 3 and 1 prior to cytolysis assay. To assay that of complement, 60 units of Cobra venom factor (Quiadel) were administrated i.p. 1 day prior to cytolysis assay and 20 units the same day of the assay, respectively.

### Uptake of SpFr and SpFr-IC by DCs

SpFr were stained with the red-fluorescing dye PKH26 (Sigma) [66] at a concentration of 2 µg/ml for 5 min and half of the preparation was used to form SpFr-IC. Fluorescent SpFr and SpFr-IC were then placed in the presence of DCs at a 1∶1 ratio for various periods of time at either 37°C, to measure uptake, or 0°C, to measure simple binding. Uptake was quantified by flow cytometry after subtraction of simple binding signals.

### Statistics

Data are presented as mean ± SEM. Statistical significance between the groups was determined by applying either the Student's t test, when two groups were compared, or a non-parametric one-way ANOVA test with Dunn's multiple comparison post-test when three groups were compared. A *P* value lower than 0,05 was considered as statistically significant.

## Supporting Information

Figure S1 A short 667 mAb treatment prevents mice from developing leukemia. Three groups of 8 day-old mice were infected with FrCas^E^. One group was treated with 667 (infected/treated) 1 hour post-infection and on days 2 and 5 post-infection, a second group was treated with a control non-neutralizing mAb of the same isotype (anti-β-2,6-fructosan IgG2a mAb from Sigma; infected/control mAb-treated), and the third group was not treated (infected/non-treated). A fourth group of mice, not infected but treated with 667, was taken as control (non-infected/treated). (A) *Survival of mice*. (B) *Hematocrits*. The presented data are the average of values obtained from at least 10 animals per time point. Error bars indicate SEM. Lines indicate linear regressions. For the sake of clarity, the percentage of hematocrit of infected/control mAb-treated mice and that of non-infected/treated mice was not shown but they were comparable to that of infected/non-treated and infected-treated, respectively. (C) *Spleen weights*. Infected/control mAb-treated- and infected/non-treated mice showed comparable spleen swelling (not shown). Bars indicate mean values. Data presented are the compilation of results from 3 independent experiments: (infected/treated mice, *n* = 29; infected/non-treated, *n* = 25 mice; non-infected/treated mice, *n* = 15; infected/control mAb-treated, *n* = 4 mice).
(0.37 MB TIF)

Figure S2 Characterization of the humoral response developing in infected/treated mice (complement to **Figure 1**). (A) *anti-FrCas^E^ IgG2a and IgG1 responses*. Anti-FrCas^E^ IgG2as and IgG1s contained in the sera of mice analyzed in **Figure 1C** were assayed by ELISA. One of 2 independent experiments with similar outcomes is presented. The data are the average of values obtained from at least 10 animals per time point. Non-infected/treated mice were negative for anti-FrCas^E^ IgGs and are not presented. Error bars indicate SEM. (B) *Neutralization activity*. Mice were infected as described in **Figure 1**. Neutralization activity was assayed by FIA in the presence of the indicated serum dilutions of pooled sera from at least 4 mice per time point and per condition. The values are the average of the results obtained in 2 independent experiments.
(0.35 MB TIF)

Figure S3 Phenotypic characterization of CD8^+^ T-cell responses in infected/treated animals (complement to **Figure 2**). Mice were infected and treated as in **Figure 1**. CD3^+^ cells were isolated by negative selection from spleens of 2 age-matched non-infected/non-treated mice and 3 infected/treated mice on day 56 post-infection. CD3^+^ cells were then stained with the D^b^-GagL tetramer and anti-CD8- and anti-CD127 mAbs for flow cytometry analysis. Data from 1 age-matched non-infected/non-treated mice and 3 infected/treated mice are presented. These data, together with the results presented in **Figure 2**, show that infected/treated mice generate a substantial CD8^+^ T-cell memory pool mainly composed of effector-memory cells.
(0.18 MB TIF)

Figure S4 Statistical analysis of the data presented in **Figure 4B**. The proliferation of CFSE-labeled GagL-TCR-TG CD8^+^ T cells was measured in each assay by quantifying the percentage of cells with decreased CFSE fluorescence (CFSE staining is divided by two at each cell division). The data from 3 independent experiments were statistically analyzed using the Student's t test (*P = 0,003).
(0.17 MB TIF)

Figure S5 (complement to **Figure 5**). *In vivo* enhancement of the anti-FrCas^E^ CD8^+^ T-cell response by cellular ICs. SpFr, or SpFr-IC, were administered i.v. to non-infected/non-treated mice. Two groups of mice (*n* = 3) were used. In one of them, 2×10^6^ FrCas^E^-infected splenocytes (SpFr) were i.v. injected in the absence of 667 whereas, in the other group, the same amount of FrCas^E^-infected splenocytes was used after immune complex formation with 150 µg of 667 (SpFr-IC). Nine days later, both the expansion of GagL-specific CD8^+^ T cells and CTL activity were assessed *in vivo* as described in **Figure 3**. Non-infected/non-treated mice with no further treatment (NI/NT) were used as controls *(A). Proliferation of GagL-specific CD8^+^ T cells*. The data obtained with the 3 mice receiving either SpFr or SpFr-IC mouse are presented together with 1 control mouse. *(B) CTL activity against GagL-loaded splenocytes*. The data obtained with the 3 mice receiving either SpFr or SpFr-IC are presented together with 1 control mouse.
(0.59 MB TIF)
