# Supplementary figures and images for: A Crucial Role for Infected-Cell/Antibody Immune Complexes in the Enhancement of Endogenous Antiviral Immunity by Short Passive Immunotherapy
Source: PLoS Pathog. 2010 Jun 10;6(6):e1000948. doi: 10.1371/journal.ppat.1000948 (PMC2883599; doi:10.1371/journal.ppat.1000948)

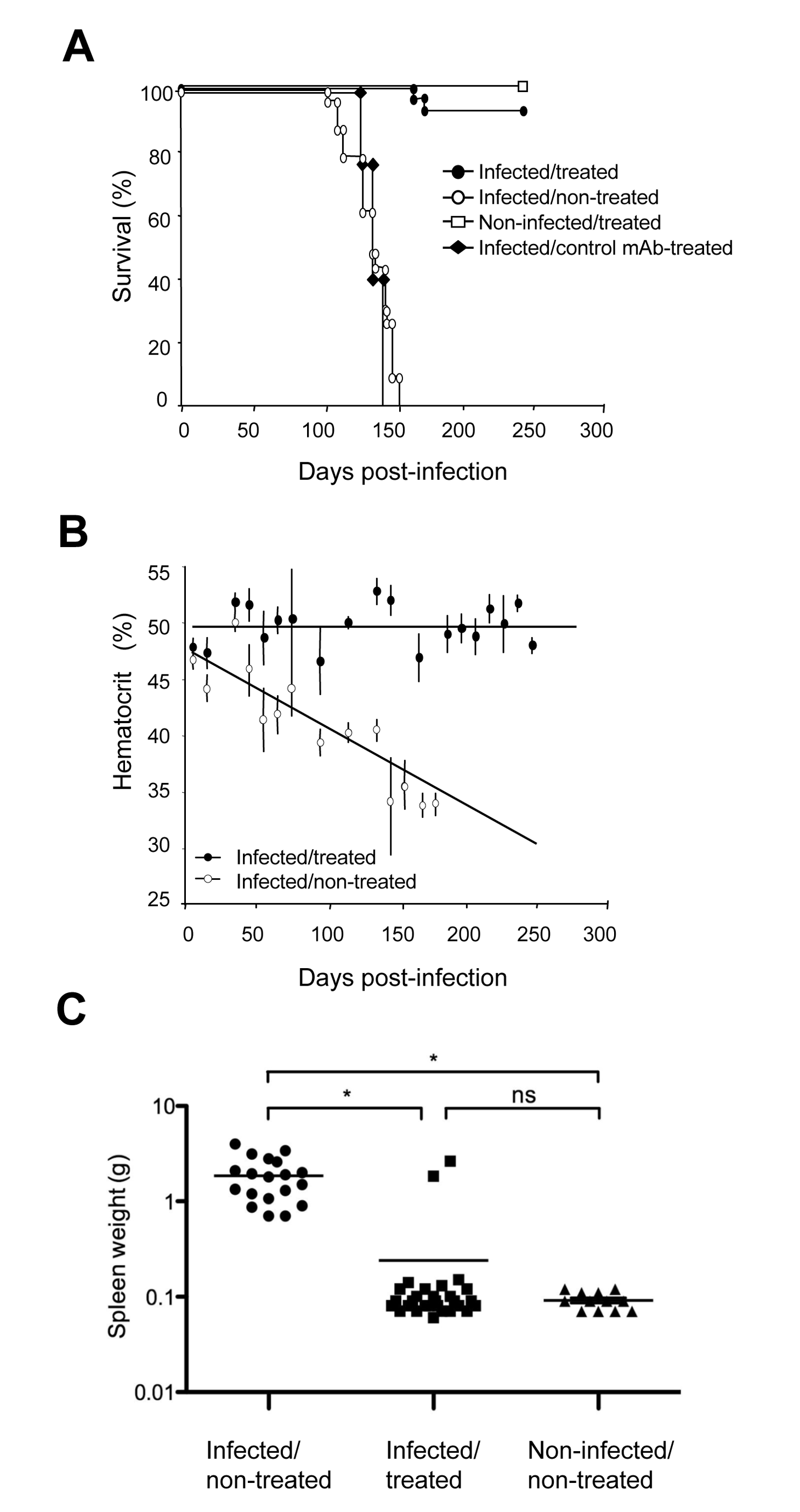

Supplement: Figure S1 — A short 667 mAb treatment prevents mice from developing leukemia. Three groups of 8 day-old mice were infected with FrCasE. One group was treated with 667 (infected/treated) 1 hour post-infection and on days 2 and 5 post-infection, a second group was treated with a control non-neutralizing mAb of the same isotype (anti-β-2,6-fructosan IgG2a mAb from Sigma; infected/control mAb-treated), and the third group was not treated (infected/non-treated). A fourth group of mice, not infected but treated with 667, was taken as control (non-infected/treated). (A) Survival of mice. (B) Hematocrits. The presented data are the average of values obtained from at least 10 animals per time point. Error bars indicate SEM. Lines indicate linear regressions. For the sake of clarity, the percentage of hematocrit of infected/control mAb-treated mice and that of non-infected/treated mice was not shown but they were comparable to that of infected/non-treated and infected-treated, respectively. (C) Spleen weights. Infected/control mAb-treated- and infected/non-treated mice showed comparable spleen swelling (not shown). Bars indicate mean values. Data presented are the compilation of results from 3 independent experiments: (infected/treated mice, n = 29; infected/non-treated, n = 25 mice; non-infected/treated mice, n = 15; infected/control mAb-treated, n = 4 mice). (0.37 MB TIF) [file ppat.1000948.s001.tif]

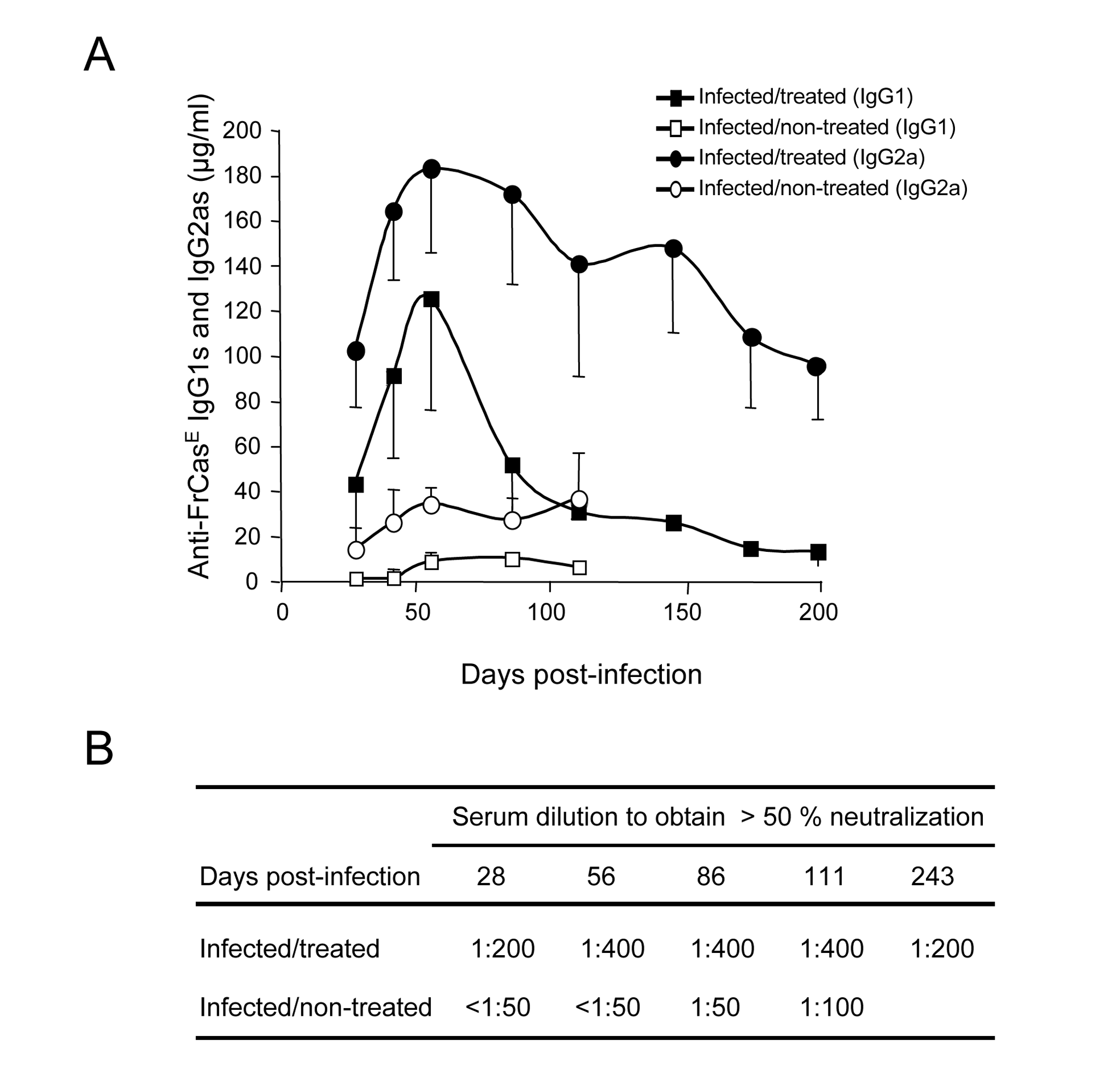

Supplement: Figure S2 — Characterization of the humoral response developing in infected/treated mice (complement to Figure 1 ). (A) anti-FrCasE IgG2a and IgG1 responses. Anti-FrCasE IgG2as and IgG1s contained in the sera of mice analyzed in Figure 1C were assayed by ELISA. One of 2 independent experiments with similar outcomes is presented. The data are the average of values obtained from at least 10 animals per time point. Non-infected/treated mice were negative for anti-FrCasE IgGs and are not presented. Error bars indicate SEM. (B) Neutralization activity. Mice were infected as described in Figure 1 . Neutralization activity was assayed by FIA in the presence of the indicated serum dilutions of pooled sera from at least 4 mice per time point and per condition. The values are the average of the results obtained in 2 independent experiments. (0.35 MB TIF) [file ppat.1000948.s002.tif]

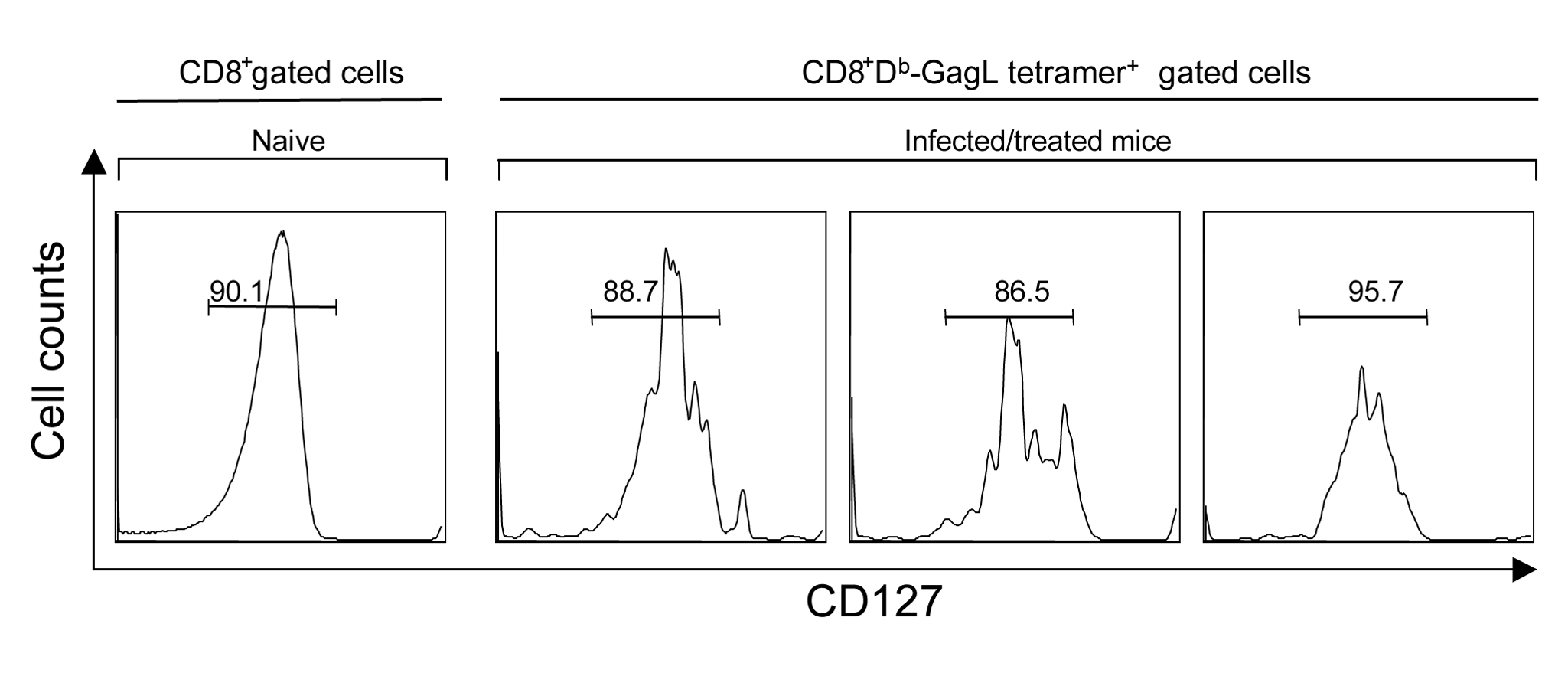

Supplement: Figure S3 — Phenotypic characterization of CD8+ T-cell responses in infected/treated animals (complement to Figure 2 ). Mice were infected and treated as in Figure 1 . CD3+ cells were isolated by negative selection from spleens of 2 age-matched non-infected/non-treated mice and 3 infected/treated mice on day 56 post-infection. CD3+ cells were then stained with the Db-GagL tetramer and anti-CD8- and anti-CD127 mAbs for flow cytometry analysis. Data from 1 age-matched non-infected/non-treated mice and 3 infected/treated mice are presented. These data, together with the results presented in Figure 2 , show that infected/treated mice generate a substantial CD8+ T-cell memory pool mainly composed of effector-memory cells. (0.18 MB TIF) [file ppat.1000948.s003.tif]

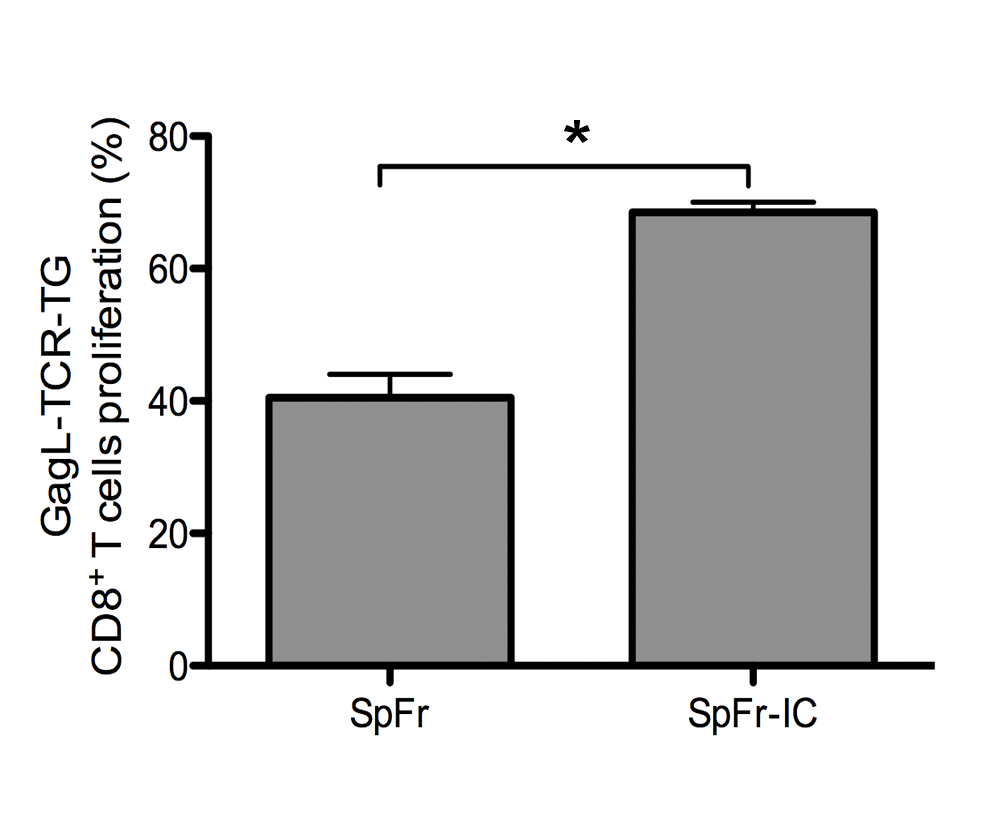

Supplement: Figure S4 — Statistical analysis of the data presented in Figure 4B . The proliferation of CFSE-labeled GagL-TCR-TG CD8+ T cells was measured in each assay by quantifying the percentage of cells with decreased CFSE fluorescence (CFSE staining is divided by two at each cell division). The data from 3 independent experiments were statistically analyzed using the Student's t test (*P = 0,003). (0.17 MB TIF) [file ppat.1000948.s004.tif]

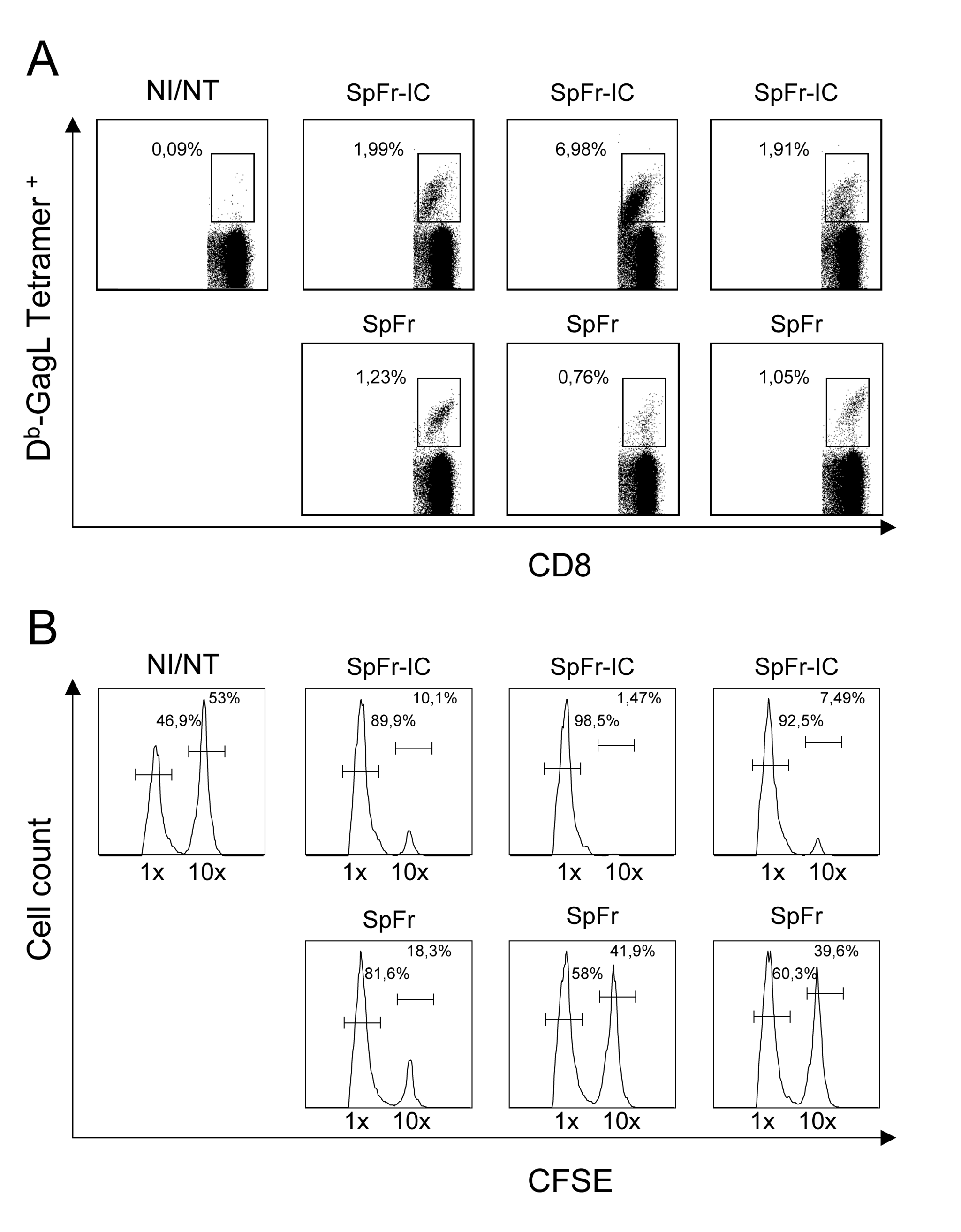

Supplement: Figure S5 — (complement to Figure 5 ). In vivo enhancement of the anti-FrCasE CD8+ T-cell response by cellular ICs. SpFr, or SpFr-IC, were administered i.v. to non-infected/non-treated mice. Two groups of mice (n = 3) were used. In one of them, 2×10^6^ FrCasE-infected splenocytes (SpFr) were i.v. injected in the absence of 667 whereas, in the other group, the same amount of FrCasE-infected splenocytes was used after immune complex formation with 150 µg of 667 (SpFr-IC). Nine days later, both the expansion of GagL-specific CD8+ T cells and CTL activity were assessed in vivo as described in Figure 3 . Non-infected/non-treated mice with no further treatment (NI/NT) were used as controls (A). Proliferation of GagL-specific CD8+ T cells. The data obtained with the 3 mice receiving either SpFr or SpFr-IC mouse are presented together with 1 control mouse. (B) CTL activity against GagL-loaded splenocytes. The data obtained with the 3 mice receiving either SpFr or SpFr-IC are presented together with 1 control mouse. (0.59 MB TIF) [file ppat.1000948.s005.tif]
